# Population pharmacokinetic-pharmacodynamic analysis of benznidazole monotherapy and combination therapy with fosravuconazole in chronic Chagas disease (BENDITA)

**DOI:** 10.1371/journal.pntd.0013522

**Published:** 2025-09-22

**Authors:** Frauke Assmus, Cintia Cruz, James A. Watson, Nicholas J. White, Ayorinde Adehin, Richard M. Hoglund, Bethania Blum de Oliveira, Fabiana Barreira, Ivan Scandale, Joel Tarning

**Affiliations:** 1 Mahidol Oxford Tropical Medicine Research Unit, Faculty of Tropical Medicine, Mahidol University, Bangkok, Thailand; 2 Centre for Tropical Medicine and Global Health, Nuffield Department of Medicine, University of Oxford, Oxford, United Kingdom; 3 Infectious Diseases Data Observatory (IDDO), University of Oxford, Oxford, United Kingdom; 4 Drugs for Neglected Disease initiative, Rio de Janeiro, Brazil; National Institutes of Health, UNITED STATES OF AMERICA

## Abstract

**Introduction:**

The currently recommended 8-week daily benznidazole regimen for Chagas disease is poorly tolerated. While shorter benznidazole monotherapy and combination regimens have been explored, the pharmacokinetic/pharmacodynamic (PK/PD) relationship remains poorly understood.

**Objectives:**

i) To describe the population pharmacokinetics of benznidazole and assess drug–drug interactions with fosravuconazole in patients with chronic Chagas disease, ii) to explore the relationship between benznidazole exposure and anti-trypanosomal treatment effects.

**Methods:**

This was a secondary analysis based on data from the previously published BENDITA study (NCT03378661), a dose evaluation trial in adults with chronic indeterminate Chagas disease (n = 210). Patients were randomized to placebo, the standard benznidazole dose (300 mg/day for 8 weeks), or lower total dose regimens (300 mg/day for 4 or 2 weeks; 150 mg/day for 4 weeks alone or combined with fosravuconazole 300 mg/week; 300 mg/week for 8 weeks plus fosravuconazole 300 mg/week). Benznidazole pharmacokinetics were evaluated using nonlinear mixed-effects modeling. The relationship between individual benznidazole exposure and the pharmacodynamic (PD) endpoint was explored using beta binomial regression. The PD endpoint (qPCR positivity) was defined as the proportion of qPCR-positive blood samples collected post-treatment over 12 months of follow-up, capturing the frequency of detectable parasitemia per patient.

**Results:**

Benznidazole pharmacokinetics were well described by a transit-absorption model with one-compartment disposition. Bioavailability was 13% lower in men than in women, and coadministration of fosravuconazole increased benznidazole clearance by 18% (both effects considered not clinically relevant). In the placebo arm, nearly all patients (97%) remained qPCR positive, with most showing qPCR positivity above 40%. Among patients receiving benznidazole, post-treatment qPCR positivity was substantially lower. In the 2-week arm, three patients had multiple positive qPCR samples (up to 43% PCR positivity). In contrast, individual qPCR positivity in the 4–8-week arms did not exceed 20% (i.e., one or no positive samples), with one non-adherent exception. The PK/PD analysis did not identify a significant pharmacokinetic driver of treatment response. While the study was not powered for between-arm comparisons, the findings suggest that lower total dose regimens (4 weeks daily or 8 weeks weekly) may provide efficacy comparable to the standard 8-week regimen.

**Conclusion:**

This study supports prior findings that the standard 8-week benznidazole regimen is excessive. Future trials using qPCR in factorial randomized designs should evaluate both treatment duration and dosing to optimize tolerability while maintaining efficacy.

## Author summary

Chagas Disease, caused by the parasite *Trypanosoma cruzi*, remains a major public health challenge, particularly in Latin America. Benznidazole and nifurtimox have been the standard treatment for the past 50 years, but they have severe limitations, notably their lengthy 8-week regimen and poor tolerability. In this study, we analyzed data from the BENDITA trial, a randomised dose evaluation trial conducted in Bolivian in patients with chronic Chagas disease, which explored short-course benznidazole regimens and combination therapy with fosravuconazole. To gain a better understanding of individual treatment responses, we investigated the relationship between benznidazole plasma exposures and the risk of parasitological recrudescence, considering dose, adherence, and variability in pharmacokinetics. We successfully described benznidazole’s pharmacokinetic behavior and showed that fosravuconazole does not have a clinically relevant impact on benznidazole’s pharmacokinetics. While benznidazole was clearly efficacious, no clear association between drug exposure metrics and antiparasitic effects was observed among the benznidazole treatment arms. Daily dosing for 4 weeks or once weekly dosing for 8 weeks with fosravuconazole appeared as effective as the standard 8-week daily course. The results suggest that the standard regimen may be excessive. Shorter treatment courses or once weekly dosing could improve adherence, tolerability, and cost-effectiveness. Future well-designed studies, ideally using factorial randomisation, are needed to identify the optimal dose, duration, and frequency of benznidazole administration which improves tolerability without compromising efficacy.

## Introduction

Chagas disease (CD) is a neglected, chronic, potentially life-threatening disease caused by the protozoan parasite *Trypanosoma cruzi* [1]. Approximately 6–7 million people are infected worldwide, with an additional 75 million at risk [1]. While the largest burden of disease is found in Latin America, increasing population mobility has spread CD to non-endemic regions, turning it into a global public health concern [2,3]. CD progresses from an often asymptomatic acute phase to a chronic phase, marked by very low parasite densities in blood [4,5]. Most individuals remain asymptomatic, but after several decades about one-third develop severe cardiac or gastrointestinal complications, leading to substantial mortality and morbidity [1,4,6,7].

Current treatment options are limited to two drugs, nifurtimox and benznidazole, which were discovered over half a century ago and are far from ideal [8]. Benznidazole is commonly used, and its mechanism likely involves nitroreduction by parasite nitroreductases, generating reactive intermediates that disrupt vital biological functions [9]. Although both drugs are highly effective during the acute phase, their efficacy in the chronic phase – particularly in adults – and their ability to halt progression vary [6,8,10,11]. Treatment is lengthy (30–60 days [12]) and often associated with adverse drug reactions (ADRs), which are more frequent in adults than children [13]. Across studies, approximately half of adult patients experienced ADRs, mostly mild to moderate, with fewer than 1.5% classified as severe [8,13]. Treatment discontinuation was reported in 10–29% of patients, primarily due to skin reactions [8,13]. However, field programs by Médecins Sans Frontières have shown that with close treatment surveillance adherence improves and most patients complete therapy, highlighting the importance of early ADR management, patient counselling, and follow-up [14,15]. Despite this, improved regimens or new treatments with better tolerability remain urgently needed [16].

Combination therapy has been proposed to enhance efficacy and tolerability by combining drugs with different mechanisms of action [17,18]. Azole antifungals such as posaconazole and fosravuconazole (a ravuconazole prodrug) inhibit *T. cruzi* sterol 14α-demethylase (CYP51), essential for membrane integrity [19]. While these ergosterol inhibitors have shown trypanostatic effects in preclinical studies [20], they failed to achieve sustained parasite clearance in monotherapy trials [21–23]. However, combining azoles with benznidazole has demonstrated additive or synergistic effects in murine models, providing a rationale for clinical evaluation [17,24,25].

Recent initiatives have focused on re-evaluating benznidazole dosing regimens, as current protocols rely on limited data [26–28], and the optimal dose, duration, and frequency remain uncertain. Some studies suggest that the standard benznidazole dose of 5–8 mg/kg/day for 60 days may be excessive, so dose reduction could improve tolerability without compromising efficacy [29–31]. Alternative strategies, such as shorter treatment durations [32–37], intermittent dosing [38–41], combination therapy [24,32], and extended treatment durations [42], have been explored in preclinical studies and clinical trials.

The BENDITA trial, sponsored by the Drugs for Neglected Diseases *initiative* (DNDi), was a phase 2 clinical trial assessing various benznidazole dosing regimens in adult patients with chronic indeterminate CD. The regimens included shorter courses, reduced daily doses, either as monotherapy or in combination with fosravuconazole, and intermittent weekly combination therapy. Efficacy and safety data for these regimens have been reported previously [32]. Although the overall incidence of adverse events was not significantly different between treatment groups, the standard 8-week regimen (300 mg daily) had the highest rate of treatment discontinuations due to adverse events. Moreover, neutropenia, leukopenia, and headaches were reported slightly more frequently in this group. All benznidazole arms demonstrated higher antiparasitic responses compared to placebo. However, the relationship between benznidazole dose, plasma exposure, and variability in treatment response remains poorly understood.

Establishing pharmacokinetic/pharmacodynamic (PK/PD) relationships in CD research is necessary to guide dosing optimization, but efforts have been hampered by the lack of validated biomarkers for clinical efficacy and parasitological cure [43,44]. Since seroconversion typically takes years to achieve, most recent trials have relied on sustained PCR negativity (usually over one year) as a binary efficacy endpoint [44]. However, PCR is limited by the low parasite densities in blood which are very close to the limit of detection (circa 1 parasite/10mL). In previous work, we have shown the limitations of binary PCR endpoints and proposed a probabilistic interpretation to address the variability in PCR results at these low parasite densities [45].

In this study, we present a PK/PD analysis of benznidazole based on data from the BENDITA study to support the evaluation of new treatment regimens. Our specific aims are i) to describe the population pharmacokinetics of benznidazole and investigate potential interactions with fosravuconazole, and ii) to explore the relationship between benznidazole exposure and parasitological responses. We use the proportion of qPCR-positive follow-up visits as a non-binary pharmacodynamic (PD) endpoint, distinguishing between single, intermittent, and consistent PCR-positivity to provide a better description of individual treatment responses. By characterizing the PK/PD relationship - while considering dose, adherence, and individual variability - we aimed to enhance our understanding of individual treatment responses and optimal antiparasitic treatment.

## Methods

### Clinical data

#### (i) Ethics statement.

The data used for population PK and PK/PD modeling originated from the BENDITA study, which has been published previously [32]. The trial was approved by the following ethics committees: Comité de ética de la Fundación Colectivo de Estudios Aplicados y Desarrollo Social (CEADES) on 30-Nov-2015, Comite de bioetica de la Facultad de Medicina Universidad Mayor de San Simón on 07-Mar-2016, and Comité de ética e investigación SEDES Chuquisaca (001/2016) on 01-Apr-2016. Further approvals were obtained from the Comité Etico de Investigación Clínica del Hospital Clínic de Barcelona (HCB/2016/0678) on 21-Sep-2016, and AGEMED (former UNIMED) – Competent Authority Bolivia (MS/UNIMED/CFN/042/2016) on 14-Apr-2016.

The trial was conducted in accordance with local regulations, the Declaration of Helsinki and Good Clinical Practice guidelines. All participants provided written informed consent prior to enrollment. The study was registered at ClinicalTrials.gov (NCT03378661, https://clinicaltrials.gov/ct2/show/NCT03378661), and the protocol is available on the DNDi website: https://dndi.org/research-development/portfolio/new-benz-regimens/.

#### (ii) Study design and study subjects.

BENDITA was a double-blind, double dummy, placebo-controlled, randomized, multicenter, dose evaluation, proof-of-concept trial conducted between 2016–2018 in three specialized Chagas disease outpatient units in Bolivia (Cochabamba, Tarija, Sucre). A total of 210 adults (18–50 years, 50–80 kg body weight) with chronic indeterminate CD, confirmed by serological testing and positive qualitative PCR results, were enrolled in the study. Subjects with chronic health conditions and signs or symptoms of the chronic cardiac or digestive form of CD were excluded.

#### (iii) Treatment regimens.

Participants were randomly assigned to one of seven treatment groups, including six active treatment arms and one placebo arm, with 30 subjects per group. The active treatment arms (180 subjects) received benznidazole as follows: benznidazole 150 mg twice daily for 8 weeks (standard care), 4 weeks, or 2 weeks; benznidazole 150 mg once daily for 4 weeks, either as monotherapy or in combination with fosravuconazole; and benznidazole 300 mg (divided in two doses) once weekly for 8 weeks in combination with fosravuconazole. Benznidazole or benznidazole-matched placebo tablets (Abarax, Laboratorios ELEA, Buenos Aires, Argentina) were administered orally in two separate doses each day (morning and evening). Fosravuconazole (Eisai Co, Ltd., Tokyo, Japan) was administered orally at a loading dose of 300 mg once daily for 3 days, followed by 300 mg once per week for 8 weeks.

#### (iv) Follow-up visits and PCR.

Blood sampling follow-up visits took place daily on days 1–3, then at weeks 2, 3, 4, 6, 10 and 12, and subsequently at 4, 6, and 12 months. At each visit, three 5 mL venous blood samples were collected and assayed in triplicate for *T. cruzi* satellite DNA, using a previously validated quantitative real-time PCR (qPCR) method [46]. Parasitological response was determined qualitatively based on qPCR results, with the overall outcome at each visit classified as ‘PCR positive’ if at least one sample tested positive. Cycle threshold (Ct) values, a measure of parasite density, represent the mean of nine measurements from three blood samples assayed in triplicate at each study visit. Higher Ct values indicate lower parasite densities (maximum reported value: 40).

#### (v) Quantification of benznidazole.

Sparse whole blood samples for pharmacokinetic analyses were obtained pre-dose and at blood sampling follow-up visits up to week 10 (n = 9 protocol time points for each patient). Precise records of drug administration timing and blood draws were utilized for population pharmacokinetic modeling.

Concentrations of benznidazole (and ravuconazole) in dry blood spots (DBS) were quantified using a validated liquid chromatography tandem mass spectrometry method (HPLC-MS/MS), conducted in accordance with Good Laboratory Practice, as described previously [47]. Briefly, intra- and inter-day precision and bias were reported to be less than 15% (n = 9) and 10% (n = 27), respectively. The lower limits of quantification LLOQ) for benznidazole and ravuconazole were 50 ng/mL and 20 ng/mL, respectively.

### Assessment of consistency in pharmacokinetic data

Initial exploration of individual benznidazole DBS concentration-time profiles revealed some inconsistencies. Some profiles had benznidazole levels which either exceeded or fell below plausible levels. For instance, benznidazole levels above the LLOQ were observed beyond 120 hours after dosing (‘TAD [time after dose] outlier’), which is not compatible with benznidazole’s reported half-life (~ 12–13 hours) [48]. As a result, a systematic and comprehensive outlier analysis was conducted (detailed in S1 Text). Additional PK outliers were identified through both the simeval and qa tools in PsN 5.2.6, supplemented by manual inspection of PK profiles. Other data sources (e.g., ADaM files, bioanalytical data) were reviewed to ensure data integrity and identify any further anomalies in PK data or uncertainties in drug exposure.

Subsequently, potential causes of these anomalies - including bioanalytical assay issues, non-adherence, physiological variability, and potential treatment misallocations - were considered to inform objective decisions regarding the inclusion or exclusion of subjects from subsequent analyses. Subjects were flagged for potential misallocation if their observed concentrations deviated markedly from the expected range for their assigned treatment, but aligned more closely with an alternative regimen. These discrepancies were identified using stochastic simulations in NONMEM, based on a preliminary population PK model. Benznidazole concentration-time profiles were simulated for 1,000 hypothetical adults per regimen, and 99% confidence intervals (CIs) were generated for each PK sampling time point. Subjects were flagged as potential treatment misallocations if they showed fewer discrepancies with an alternative treatment arm than with their assigned one. Documented non-adherence and treatment interruptions were considered. These participants, where treatment misallocation could not be ruled out, were excluded from all analyses.

### Population pharmacokinetic analysis

#### (i) Model development.

All subjects allocated to active treatment arms were included in the final PK analysis dataset, except those flagged for potential treatment misallocation. Sparse DBS concentrations of benznidazole were pooled across all six active treatment arms. DBS concentrations were transformed into their natural logarithms, and concentration-time profiles modeled using nonlinear mixed-effects modeling in NONMEM, version 7.4.3 (Icon Development Solution, Ellicott City, MD, USA). The first-order conditional estimation method with interactions (FOCE-I) was employed. The model building process and model diagnostics were facilitated by the use of Pirana (v2.9.9), Pearl-speaks-NONMEM (PsN v5.2), R (v4.2.2) and TIBCO Spotfire (v 11.3.0) [49,50]. Discrimination between two competing, nested models was based on objective function values (OFV), with the difference in OFV being equivalent to a likelihood ratio test. A significant improvement of the structural model was indicated by a decrease of OFV by at least 3.84 (p < 0.05, one degree of freedom difference). Pharmacokinetic data were censored at 120 hours TAD (approximately 10 benznidazole half-lives), and concentrations below the LLOQ for the censored dataset were omitted.

One- and two compartment disposition models were evaluated, along with different absorption models (first-order absorption and transit compartment models). Relative bioavailability (F) was fixed to unity for the population, as all data were from oral administration and no intravenous data were available to estimate absolute F. Inter-individual variability (IIV) on F was incorporated into the base model, and covariate effects - such as sex - were modeled as relative differences in bioavailability. IIV was implemented using an exponential model, with IIV estimates below 10% fixed to zero in the final model. Residual unexplained variability was implemented as an additive error on the log-transformed observed concentrations (equivalent to an exponential residual error on an arithmetic scale).

The best performing structural model was carried forward to subsequent covariate model building. Body weight (standardized to a body weight of 65 kg) was implemented a priori as an allometric function on all clearance (exponent 0.75) and volume (exponent 1) parameters. Various additional demographic, pharmaceutical and parasitological factors were evaluated based on statistical significance and biological plausibility. Individual creatinine clearance was estimated from blood creatinine levels using the Cockcroft and Gault equation. Preselected covariates (age, sex, accumulated benznidazole dose and dose per day, duration of drug treatment, monotherapy/combination with fosravuconazole, markers of liver/kidney function, hematocrit, and serology) were further evaluated by a step-wise covariate modeling approach, using the scm functionality in PsN. For continuous covariates, linear, exponential, and power parameter-covariate relationships were explored. In the forward step, covariates were included at a statistical significance level of p = 0.05 (ΔOFV = −3.84), followed by a more stringent backward elimination step (p = 0.001, ΔOFV= −10.83).

Basic goodness-of-fit (GOF) diagnostics were used to identify potential model misspecifications and systematic errors. The predictive performance of the final model was assessed by prediction-corrected visual predictive checks (VPCs). Bootstrapping (n = 1,000 resampled bootstrap datasets) was performed to evaluate model robustness and obtain parameter precision estimates.

#### (ii) Definition of exposure metrics.

The following individual PK exposure metrics were extracted: peak concentrations (C_MAX_) and areas under the concentration-time curves to infinity (AUC_∞_) for benznidazole in blood. The time above the putative minimum target concentration (T > IC_90_) was estimated, with ${IC}_{\text{9}\text{0},DBS}$ for benznidazole in blood set at 29.3 µM (7.61 mg/L). This target concentration is based on the in vitro ${IC}_{\text{9}\text{0}}$ of benznidazole against the amastigote form of *T. cruzi* (Tulahuen strain) in 3T3 host cells, corrected for protein binding and scaled to human blood (DBS) samples. Further details are provided in S2 Text. A sensitivity analysis was also performed using previously suggested therapeutic ranges as thresholds (3–6 mg/L in plasma or 2.5-5 mg/L in DBS), instead of the IC_90_.

In addition to these exposure metrics, the duration of benznidazole treatment was described in several ways: total days of benznidazole treatment (actual number of days benznidazole was taken); weeks of benznidazole treatment (a week is counted if at least one benznidazole dose per week was taken); and total duration of benznidazole treatment, irrespective of intermittent drug interruptions. For example, if an individual randomised to 2 weeks benznidazole took active treatment only on days 1 and 12, this would be recorded as 2 total days of treatment, 2 weeks of treatment, and 12 days total duration of treatment.

### Assessment of treatment failure

In this retrospective analysis, any positive *T. cruzi* qPCR result at the end of treatment (EOT) or during any follow-up post-EOT was considered indicative of treatment failure. EOT was defined according to the assigned treatment duration, allowing for a two-week grace period after the last active dose. Treatment failure was illustrated in both the modified intention-to-treat (mITT) and per-protocol (PP) populations. The mITT population included all participants with post-EOT follow-up data, excluding those with potential treatment misallocation. The PP population further excluded individuals with <80% benznidazole compliance. Compliance was assessed at the level of individual dosing occasions, accounting for partial dosing (e.g., one dose taken instead of two on a given day).

The proportion of patients with at least one qPCR-positive sample post-treatment was summarized per arm, with Clopper-Pearson 95% CIs. Fisher’s exact test (Holm-adjusted p-values) was used to compare active arms to placebo. As the study was not powered to differentiate between active treatment arms, no formal statistical comparisons were performed between them. Unlike the original BENDITA study, which standardized the number of follow-up visits across treatment arms by excluding specific visits (e.g., two in the 2-week arm), our analysis retained all available post-treatment qPCR data to support PK/PD modeling, specifically for the subsequent calculation of qPCR positivity (see below).

### Exposure - parasitological response analysis

To assess the exposure–response relationship, a non-binary PD endpoint (qPCR positivity) was used, defined as the proportion of *T. cruzi* qPCR-positive blood samples post-EOT. qPCR positivity was calculated for each subject as (Equation 1):


$$qPCR\;positivity\;=\frac{number\;of\;follow-up\;visits\;with\;positive\;qPCR\;samples}{total\;number\;of\;follow-up\;visits}\times \text{1}\text{0}\text{0}\;\%\;$$ (1)

The analysis was conducted in the modified intention-to-treat (mITT) population. Participants without post-EOT follow-up or with potential treatment misallocation were excluded but not considered treatment failures, as their post-treatment status could not be assessed. Excluding these individuals ensured the analysis focused on participants with reliable exposure and outcome data. Sensitivity analyses excluding additional PK outliers and individuals with uncertain exposure were conducted to assess the robustness of findings.

The relationship between benznidazole exposure in blood and qPCR positivity in the mITT population was explored using beta binomial regression in R (v4.2.2).

The model was specified as follows:


$$logit(p)={\text{log}\left(\frac{p}{\text{1}-p}\right)=\beta }_{\text{0}}+\beta_{\text{1}}x+\beta_{\text{2}}Ct,\;$$ (2)

where *p* represents the probability that a qPCR sample is positive for the presence of parasites at a single follow-up visit, and logit (*p*) represents the log odds of qPCR positivity. In Equation 2, β_0_ is the intercept, while β_1_ and β_2_ represent the change in the log-odds ratio for qPCR positivity per unit increase in the predictor variable x and the Ct value at the screening visit (pre-dose), respectively. Various exposure variables (*x*) were investigated as predictors of the PCR positivity, including individual C_MAX_, AUC_∞_, T > IC_90_, and benznidazole treatment durations.

The probability of at least one follow-up sample being qPCR positive, denoted as p*, was estimated from the probability of qPCR positivity for a single follow-up visit (p), as follows:


$$p^{*}=\text{1}-(\text{1}-p{)}^{N},\;$$ (3)

where N is the number of follow-up visits. Predictions were made for a range of follow-up visits, and utilizing the median Ct value at baseline.

Model implementation was conducted using the ‘glmmTMB’ package [51], with the beta-binomial distribution parameterized according to Morris 1997 [52]. After excluding placebo data and one subject who only took 4 doses over 8 days, a subgroup analysis was performed using binomial regression modeling in the GLM family in R. This subgroup analysis did not support a beta binomial model and assumed a dispersion factor of 1. Model diagnostics, including tests for overdispersion and zero inflation, as well as simulated residuals, were performed using the ‘DHARMA’ package in R [53]. Model comparisons were based on the Akaike information criterion (AIC).

## Results

### Characterization of the study population

**Fig 1:**
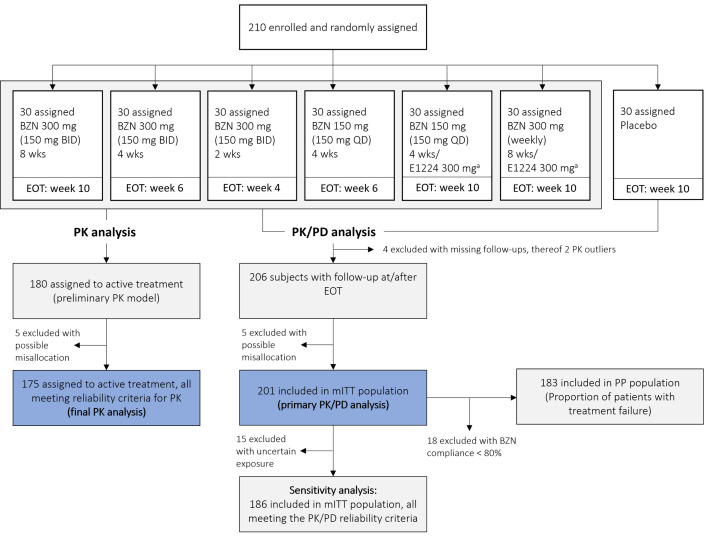
Flow diagram showing the number of patients randomised to each group, and exclusions from the PK and PK/PD analyses. Dark blue boxes indicate the primary analysis populations for the final PK and PK/PD analyses, following the exclusion of subjects with significant discrepancies between observed and predicted benznidazole concentrations, which may be explained by treatment misallocations, though the exact cause remains uncertain (n = 5). For the PK/PD sensitivity analysis, all subjects not meeting the PK/PD reliability criteria (PK outliers or uncertain exposure) were excluded from the mITT population, resulting in restricted population of 186 subjects. **Abbreviations:** EOT, end of treatment; BZN, benznidazole; E1224, fosravuconazole; wks, weeks; QD, once daily; BID, twice daily; mITT, modified intention-to-treat; PP, per-protocol.

A total of 210 adults with chronic indeterminate CD were enrolled in the BENDITA study, with 30 patients randomly assigned to each treatment group (Fig 1). Prior to analysis, a systematic assessment of PK data consistency was conducted to evaluate its reliability. Details of inconsistencies by diagnostic criteria and likely causal factors are provided in S1 Text (S1.2 and S1.3). For most subjects, the specific reasons for these discrepancies remain unclear. However, five subjects exhibited significant discrepancies between observed and predicted benznidazole concentrations, warranting their exclusion from all analyses. Treatment allocation errors were one possible explanation, but other factors - such as incorrect sample labelling, non-adherence, or assay errors - cannot be ruled out.

The final PK analysis included 175 subjects allocated to active treatment. Four patients had no follow-up post-EOT and were excluded, leaving 201 patients in the mITT population (including placebo). The PP population comprised 183 patients after excluding those with <80% benznidazole compliance. For the PK/PD analysis, a sensitivity analysis was performed in a subset of 186 patients from the mITT population, excluding a total of 22 subjects with uncertain drug exposure who did not meet the PK/PD reliability criteria (e.g., PK outliers not attributable to an assay error). Further details on the exclusion criteria are provided in S1 Text (S1.4). A detailed flow chart of patient exclusions by treatment arm is provided in S1 Fig.

Baseline characteristics and adverse events for the entire BENDITA study population have been reported previously [32]. Here, demographic information for subjects included in the PK and PK/PD analyses is presented (Table 1). All patients were Bolivian and aged between 18 and 50 years, with a median body weight of 65 kg (range 50–80 kg). The majority of patients were female (70% female, 30% male, mITT). Demographic and clinical characteristics were similar across treatment arms.

**Table 1:** Summary of baseline characteristics, compliance and adverse events affecting exposure of the study population.

Characteristic	BZN 300 mg daily for 8 weeks (n = 30)	BZN 300 mg daily for 4 weeks (n = 29)	BZN 300 mg daily for 2 weeks (n = 29)	BZN 150 mg daily for 4 weeks (n = 29)	BZN 150 mg daily for 4 weeks + E1224 (n = 29)	BZN 300 mg once weekly for 8 weeks + E1224 (n = 29)	Placebo (n = 30)	PK analysis dataset (n = 175)	PK/PD (mITT) analysis dataset (n = 201)
Sex: male, n (%)	11 (36.7%)	8 (27.6%)	8 (27.6%)	11 (37.9%)	11 (37.9%)	7 (24.1%)	5 (16.7%)	56 (32%)	60 (29.9%)
Age, years	31.5 (20,47)	33 (18,50)	33 (18,46)	36 (20,48)	29 (18,48)	34 (22,49)	32 (21,50)	34 (18,50)	33 (18,50)
Weight, kg	68 (51.0,78.0)	65.3 (52.0,75.7)	62.5 (50.0,79.0)	64.4 (53.0,80.0)	65 (50.0,79.2)	67.5 (51.0,80.0)	63.3 (51.0,79.0)	65 (50.0,80.0)	64.5 (50.0,80.0)
Body mass index, kg/m^2^	27.9 (20.7,34.1)	25.8 (21.4,31.9)	24.8 (20.3,29.9)	26.5 (21.0,35.1)	24.8 (21.1,38.2)	26.1 (21.0,38.6)	26.8 (21.2,33.9)	25.9 (20.3,38.6)	26.1 (20.3,38.6)
Alkaline Phosphatase, IU/L (normal: 30–120 IU/L)^a^	195 (50.0,339)	154 (52.0,329)	190 (51.0,311)	208 (58.0,282)	214 (56.0,317)	201 (51.0,321)	211 (57.0,310)	195 (50.0,339)	199 (50.0,339)
Alanine Amino-transferase, IU/L (normal: 10–40 IU/L)^a^	22.7 (11.0,39.0)	20.2 (14.0,42.0)	21.5 (10.6,34.4)	23 (11.7,46.0)	25 (10.9,46.0)	23 (9.00,41.0)	27.3 (13.0,72.0)	22 (9.00,46.0)	23 (9.00,72.0)
Aspartate Amino-transferase, IU/L (normal: 10–40 IU/L)^a^	22 (14.0,32.0)	25 (12.0,43.7)	22 (14.4,40.0)	23.7 (13.0,37.0)	23 (13.0,35.3)	22 (13.0,36.8)	23.0 (13.0,58.0)	23 (12.0,43.7)	23 (12.0,58.0)
Bilirubin, total, umol/L (normal:5.13-17.1 umol/L)^a^	12.1 (6.84,20.5)	14.9 (8.6,20.5)	14.9 (7.36,20.5)	13.7 (6.84,20.5)	15.4 (6.84,20.5)	12.0 (8.55,20.5)	13.7 (6.84,20.5)	13.7 (6.84,20.5)	13.7 (6.84,20.5)
Creatinine Clearance, mL/min (normal:90–120 mL/min)^b^	103 (69.5,180)	94 (67.5,152)	97.4 (54.8,135)	103 (72.0,150)	104 (65.3,150)	108 (64.3,162)	103 (77.1,176)	102 (54.8,180)	103 (54.8,180)
Baseline Ct	38.2 (31.1, 40)	36.7 (32.0, 40.0)	38.3 (33.4, 39.9)	37.6 (32.2, 39.5)	38.1 (33.3, 39.9)	37.6 (30.2, 39.8)	37.9 (31.0, 39.9)	37.7 (30.2 - 40.0)	37.7 (30.2, 40.0)
Compliance									
• Compliant subjects (all), n (%)	24 (80.0%)	27 (93.1%)	28 (96.6%)	28 (96.6%)	24 (82.8%)	26 (89.7%)	30 (100%)	157 (89.7%)	186 (92.5%)
• Compliant subjects (BZN), n (%)	24 (80.0%)	27 (93.1%)	23 (79.3%)	27 (93.1%)	27 (93.1%)	27 (93.1%)	–	155 (88.6%)	–
Adverse events affecting exposure to treatment									
• Leading to interruption, n(%)	5 (17%)	7 (24%)	10 (34%)	5 (17%)	3 (10%)	1 (3%)	(3%)	31 (18%)	31 (15%)
• Leading to discontinuation, n(%)	6 (20%)	1 (3%)	0	1 (3%)	3 (10%)	4 (14%)	0	15 (9%)	15 (7%)

All values are given as median (minimum, maximum range), unless otherwise indicated. Subjects with severe PK outliers, which may be indicative of treatment misallocation (n = 5), were excluded from all datasets. Subjects with missing follow-up post-EOT were excluded from the PK/PD (mITT) analysis population. Compliant subjects (all) were defined as those who took ≥80% of their allocated treatment (benznidazole, E1224, or placebo). For BZN-specific compliance, this refers to ≥80% of the allocated benznidazole dose. **Abbreviations:** BZN, benznidazole; E1224, fosravuconazole; mITT, modified intention-to-treat.

^a^Normal ranges from American Board of Internal Medicine (January 2024);

^b^Normal ranges taken from [54].

The proportion of adherent subjects (those who took at least 80% of their allocated benznidazole dose) exceeded 90% in most study arms, except for the 8-week and 2-week treatment arms, where adherence was approximately 80%. As reported previously, treatment discontinuations due to adverse events were most common in the 8-week group (20%), followed by the combination therapy arms. Treatment interruptions due to adverse events were most frequent in the 2-week group (34%), but there were no treatment discontinuations in this group.

### Pharmacokinetics

The 175 patients included in the pooled population PK analysis contributed 986 DBS benznidazole concentrations, of which 969 were above the LLOQ (1.7% BQL).

**Fig 2:**
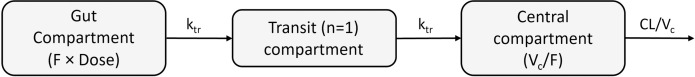
Graphical representation of the structural model describing the pharmacokinetics of benznidazole in the BENDITA study. Absorption from the gut compartment is described by a transit absorption model (with one transit compartment), followed by a one-compartment disposition model. F is the relative oral bioavailability, V_C_ is the apparent volume of distribution of the central compartment, and CL is the apparent elimination clearance. k_TR_ is the rate constant between absorption compartments and was calculated from the mean absorption transit time (MTT) as k_TR_ = (1 + n)/MTT where n is the number of transit compartments.

Benznidazole concentration-time profiles were best described by a transit-compartment absorption model, followed by a one-compartment disposition model, with linear elimination from the central compartment (Fig 2). A two-compartment disposition model did not significantly improve the model fit. A transit-compartment absorption model with one transit compartment was superior to all other absorption models.

Body weight was included as an allometric function on CL/F (exponent = 0.75) and Vc/F (exponent = 1.0), resulting in a substantial model improvement (∆OFV = -34.7). A potential time-dependency of CL/F was investigated using linear, exponential, and E_MAX_ functions as described previously [55]. However, none of the models with time-varying clearance yielded a significant improvement in model fit. Co-administration of fosravuconazole resulted in an increase in benznidazole clearance by 18% (∆OFV = -15.7). Moreover, there was a trend towards lower relative bioavailability in men compared to women, with an estimated 13% lower bioavailability (F) in men (∆OFV = -15.5). None of the other covariates tested were retained after the backward elimination covariate step.

**Fig 3:**
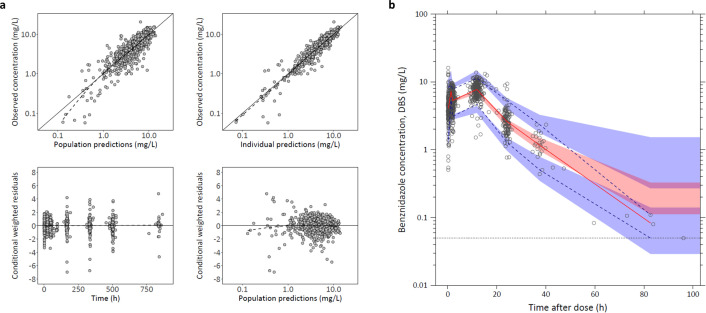
Model diagnostics of the final population PK model for benznidazole in the PK analysis population (n = 175). **a**) Goodness-of-fit. Observations are represented by grey circles. Solid black lines represent the line of identity (upper panel) or zero line (lower panel), and the dashed lines represent the local polynomial regression fitting for all observations. **(b)** Prediction-corrected visual predictive check. Open circles represent observed plasma benznidazole concentrations. The solid red line represents the 50th percentile (median) and the blue dashed lines represent the 5th and 95th percentiles of the observed data. The shaded area represents the 95% CI around the simulated 5th, 50th, and 95th percentiles.

Parameter estimates from the final model, along with their relative standard errors and confidence intervals, are provided in Table 2. The variability in mean transit time, MTT, was high (62% coefficient of variation, CV). However, bootstrapping analysis indicated a robust PK model, demonstrating moderate to high precision in estimating PK parameters (RSE < 32% for all parameters). GOF plots and the prediction-corrected VPC are shown in Fig 3. The final model accurately described the observed concentration-time profiles, with no major model misspecifications. A sensitivity analysis, re-including the five subjects with severe PK outliers, showed minimal changes in PK parameter estimates, confirming the robustness of the PK model despite potential misallocations (S1 Text, S1.5).

**Table 2:** Parameter estimates of the final population PK model of benznidazole.

Parameter	Population estimate^a^ (%RSE)^b^	Bootstrapping 95% CI^b^	IIV, %CV^a^ (%RSE)^b^	Bootstrapping 95% CI^b^
***Pharmacokinetic parameters***				
Relative oral bioavailability, F	1 *fixed*	–	10.2 (31.9)	2.97 – 15.5
Mean transit time, MTT (h) ^c^	0.75 (9.1)	0.62 - 0.89	61.6 (15.6)	35.0 – 75.3
Apparent clearance, CL/F (L/h)	1.30 (2.7)	1.24 - 1.38	18.9 (16.8)	12.6 – 25.3
Apparent volume of distribution, V/F (L)	31.6 (2.9)	29.9 - 33.5	–	–
Variance of residual error, σ	0.076 (9.3)	0.051 - 0.106	–	–
***Covariate effects:***				
Co-administration of E1224 on CL/F (%)	17.7 (28.9)	8.18 - 27.3	–	–
Sex effect on F (reference: female) (%)	-12.9 (23.0)	-18.6 - -6.9	–	–

Population estimates are given for a female adult weighting 65 kg.

^a^Population mean parameter estimates and interindividual variability (IIV) calculated by NONMEM. The coefficient of variation (% CV) for the IIV was calculated from the variance (ω^2^) as $\text{1}\text{0}\text{0}\times \sqrt{e^{\omega^{\text{2}}}-\text{1}}$.

^b^Precision of parameter estimates, based on nonparametric bootstrap diagnostics of the final PK model. Relative standard error (RSEs,%) are calculated as $\text{1}\text{0}\text{0}\times \frac{standard\;deviation}{mean\;value}$. The 95% confidence intervals (CIs) are based on the 2.5^th^ –97.5^th^ percentiles of the bootstrap parameter estimates.

^c^The mean transit time (MTT) was estimated based on a transit-compartment absorption model (with one transit compartment), which accounts for delayed absorption [56].

Benznidazole showed rapid absorption with a median time to maximum concentration (T_MAX_) ranging from 1.7 to 2.1 hours. The combination therapy arms demonstrated a slightly shorter elimination half-life (~14 hours) compared to the monotherapy arms (~17 hours), attributable to the effect of fosravuconazole co-administration on benznidazole clearance. AUC_∞_ and C_MAX_ values were lower in men compared to women, reflecting a lower relative bioavailability, but this effect was minor (<20%). Secondary PK parameter estimates for benznidazole across the different active treatment arms, stratified by sex, are provided (S1 Table).

**Fig 4:**
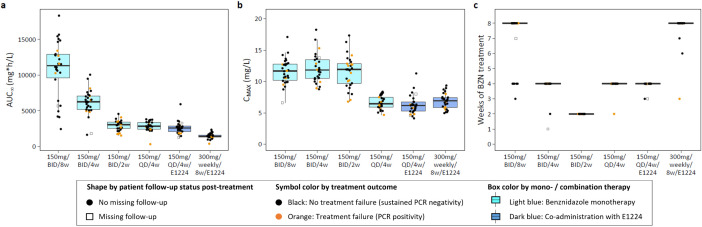
Distribution of selected exposure variables for benznidazole across treatment arms in the PK analysis population (n = 175). Boxplots display the distribution of benznidazole exposures in terms of **a)** AUC_∞_, **b)** C_MAX_, and c) weeks of treatment (a week is counted if at least one dose of benznidazole was taken). Median (midline), interquartile range (IQR, box), and 1.5*IQR whiskers are shown. **Abbreviations:** BZN, benznidazole; QD, once daily; BID, twice daily; E1224, fosravuconazole.

The distribution of selected exposure variables, not stratified by sex, is illustrated in Fig 4. Total exposure varied widely across treatment arms, with the standard treatment arm (300 mg benznidazole daily for 8 weeks) showing a 7.9-fold higher median AUC_∞_ compared to the weekly arm. AUC_∞_ exhibited approximate dose-linearity, with an approximate 2-fold increase when comparing 2 weeks to 4 weeks, and 4 weeks to 8 weeks of treatment in the monotherapy arms (300 mg daily). C_MAX_ was about twofold higher in the twice-daily treatment arms (11.7 to 11.9 mg/L) compared to the once-daily or weekly treatment arms (6.2 to 6.9 mg/L).

Apart from AUC_∞_ and C_MAX_, the duration of treatment was assessed using different definitions (S2a Fig). Duration in weeks (counting any week with at least one dose) was highly correlated with total treatment duration (i.e., duration, irrespective of interruptions). However, in the weekly dosing arm, there was a clear difference between these parameters and the total number of days benznidazole was actually taken (see S2 Table for correlation matrix of exposure metrics). For consistency, treatment duration in terms of weeks is used throughout (Fig 4c).

**Fig 5:**
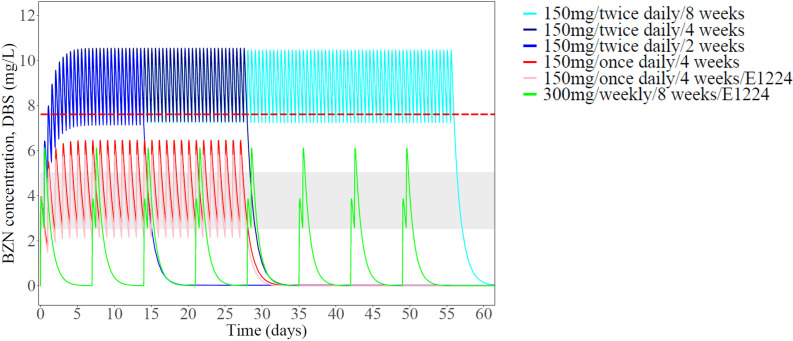
Simulated median benznidazole concentrations in DBS versus time for the BENDITA treatment arms. Simulations were performed for 2000 patients (1000 women and 1000 men, body weight: 65 kg) using the final population PK model. The horizontal red line denotes the in vitro IC_90_, adjusted for protein binding and scaled to DBS concentrations. The grey shaded area marks the historically accepted therapeutic range: 3-6 mg/L in plasma, scaled to 2.5-5 mg/L in DBS samples.

Additionally, time above the target concentration was derived, showing high sensitivity to the choice of the threshold (S2b Fig). Using the lower bound of the previously suggested therapeutic range (3 mg/L in plasma, i.e., 2.5 mg/L in DBS), extended exposure above target concentrations was achieved in all treatment arms. In contrast, using the in vitro IC_90,DBS_ (derived from activity against amastigotes), only twice-daily dosing regimens maintained levels above the target concentration. This is further illustrated in Fig 5, showing simulated median plasma concentration-time profiles for the different treatment arms. Simulated PK profiles, accounting for IIV and stratified by sex, are provided in S3 Fig.

### Proportion of patients with treatment failure

**Fig 6:**
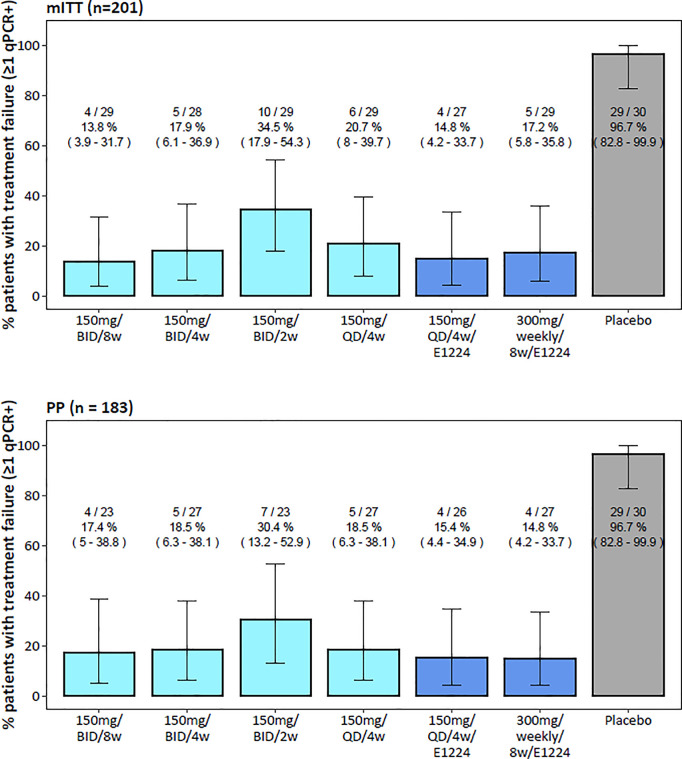
Proportions of patients with treatment failure (at least one post-treatment qPCR-positive result) in the mITT (n = 201) and PP population (n = 183). Color of barplots by benznidazole monotherapy (light blue), co-administration of E1224 (dark blue), or placebo (grey). Numbers above bars indicate patients with treatment failure/ total patients, along with the proportion of patients with treatment failure (%) and 95% Clopper Pearson CIs. No formal statistical comparisons were made between active treatment arms due to small sample sizes and differences in the number of follow-up visits.

Fig 6 presents the proportions of patients with at least one qPCR-positive result post-EOT (i.e., ‘treatment failure’) in both the mITT (n = 201) and PP (n = 183) populations. In both populations, treatment failure was significantly more frequent in the placebo arm (97%, 95% CI: 83–100, n = 29) compared to all active treatment arms (p < 0.0001). In the PP population, proportions were similar (15–19%) across most active treatment arms, except for the 2-week benznidazole regimen, where 30% (95% CI: 13–53, n = 20) had at least one positive PCR result post-treatment. Although the 2-week regimen showed a numerically higher failure rate, the 95% CIs across active treatment arms overlapped. No formal statistical comparisons were made between active regimens due to small sample sizes. Note that these proportions are based on all available follow-up samples, unlike the original BENDITA study, which excluded selected follow-ups to standardize visit numbers.

Importantly, the binary endpoint ‘treatment failure’ is influenced by the number of follow-up visits - which varied by treatment arm (S4 Fig) – and was therefore used descriptively only, not for PK/PD modeling. Instead, it served to illustrate the proportion of patients with any detectable *T. cruzi* DNA post-treatment in a compliant population (PP) and in the PK/PD modeling population with available follow-up (mITT).

### qPCR positivity: PD endpoint for PK/PD modeling

**Fig 7:**
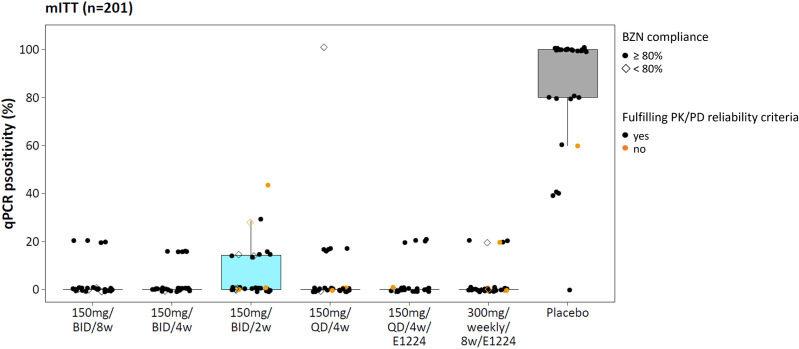
qPCR positivity proportions in the modified ITT (mITT) population (n = 201). Each dot represents an individual’s proportion of post-treatment qPCR-positive blood samples (‘qPCR positivity’). Symbol color indicates whether patients met all PK/PD reliability criteria (black) or had problematic PK data or uncertain drug exposure (orange). Closed symbols indicate ≥80% benznidazole compliance; open symbols indicate <80% compliance.

For PK/PD modeling, the proportion of qPCR-positive samples post-treatment (‘qPCR positivity’) was used as a non-binary PD endpoint in the mITT population (n = 201, Fig 7). This endpoint captured the frequency of detectable parasitemia per patient while avoiding bias due to differing numbers of follow-up visits across treatment arms. Unlike the PP analysis, participants with <80% benznidazole compliance were retained, as partially adherent individuals still provide valuable exposure–response information. Trends in qPCR positivity were consistent with the binary endpoint.

In the mITT population, 29 of 30 patients in the placebo group had at least one positive qPCR result post-treatment. Among these, qPCR positivity was consistently high (≥ 40%) in all but one patient - i.e., at least 2 of 5 follow-up samples were positive. In contrast, patients in the 4- or 8-week treatment arms were either persistently negative or had only one single PCR-positive sample, corresponding to qPCR positivity ≤20%. An exception was observed in the 4-week once-daily treatment arm, where a patient discontinued treatment after 4 doses (over 8 days) and remained qPCR-positive in all follow-up visits.

In the 2-week arm, 10 of 29 patients had at least one positive sample post-treatment. Among these, three had multiple positive qPCRs, with qPCR positivity proportions of 29% and 43% (i.e., 2 or 3 positive qPCRs out of 7 follow-up visits). These values were higher than those observed in patients receiving longer benznidazole regimens. Two of these patients were likely non-adherent, or there may have been a labeling error or placebo mix-up, and they did not meet PK/PD reliability criteria. Caution is warranted in interpreting these results. However, notably, one fully adherent patient in the 2-week treatment arm showed a qPCR positivity of 29% (2/7 positive qPCRs).

### Exposure - parasitological response model (PK/PD analysis)

**Fig 8:**
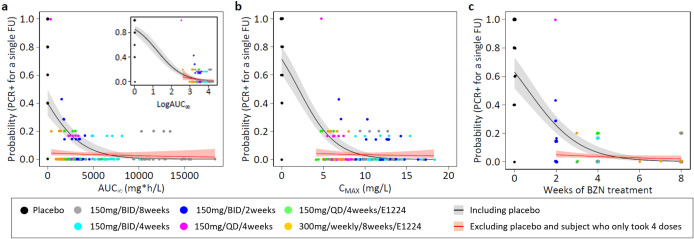
Predicted probabilities of qPCR positivity for a single follow-up visit, based on (beta) binomial regression. Model fits are shown for the modified ITT population, including placebo (n = 201, grey lines and grey shaded area) and excluding placebo as well as one influential outlier (n = 170, red lines and red shaded area). The solid grey and red lines indicate the median, and the shaded areas represent the 95% CI around predicted probabilities. Points represent observed proportions of positive qPCR results after end of treatment (qPCR positivity) in the placebo arm (black) and active treatment arms. **a)** AUC_∞_, **b)** C_MAX_, **c)** weeks of treatment (a week is counted if at least one dose of benznidazole was taken). The insert in a) shows regression based on log-transformed AUC_∞_ (an increment of 1 was added to all AUC_∞_ values to allow for log-transformation of placebo data). The influential outlier, who took only four doses of 150 mg benznidazole over 8 days, was allocated to the 150 mg benznidazole once daily for 4 weeks treatment arm and is represented by the pink point with 100% qPCR positivity.

Fig 8 presents observed qPCR positivity proportions, along with model-predicted probabilities of a positive qPCR result at a single follow-up visit for selected benznidazole exposure variables. Detailed diagnostics for this univariate (beta) binomial regression analysis are provided in Table 3.

**Table 3:** Univariate regression model diagnostics for the correlation between benznidazole exposure and qPCR positivity (mITT population).

Characteristic	Base model	AUC_∞_ (mg×h/L)	Log AUC_∞_ (mg×h/L)	C_MAX_ (mg/L)	Time above target (days)^c^		Duration of BZN treatment		
					3 mg/L in plasma	6 mg/L in plasma	Total days of dosing^d^	Weeks ^e^	Total duration (days)^f^
***Modified ITT, including placebo (n = 201)*** ^a^									
Odds ratio, exposure		0.9995	0.236	0.640	0.883	0.946	0.905	0.449	0.888
[95% CI]	–	[0.9994, 0.9997]	[0.194, 0.288]	[0.581, 0.706]	[0.851, 0.915]	[0.920, 0.973]	[0.878, 0.932]	[0.366, 0.550]	[0.862, 0.914]
p-value		<0.001***	<0.001***	<0.001***	<0.001***	<0.001***	<0.001***	<0.001***	<0.001***
Odds ratio, Ct value	0.911	0.9021	0.815	0.896	0.885	0.906	0.885	0.803	0.803
[95% CI]	[0.809, 1.027]	[0.8024, 1.0142]	[0.721, 0.920]	[0.801, 1.003]	[0.789, 0.993]	[0.804, 1.022]	[0.786, 0.995]	[0.709, 0.911]	[0.709, 0.910]
p-value	0.127	0.085	<0.001***	0.055	0.037*	0.109	0.042*	<0.001***	<0.001***
**AIC**	**439.2**	**399.7**	**283.0**	**343.5**	**372.6**	**418.3**	**382.0**	**350.3**	**348.2**
***Excluding placebo and one subject who took only 4 benznidazole doses (n = 170)*** ^b^									
Odds ratio, exposure		0.9999	0.381	0.966	0.983	0.991	0.986	0.854	0.975
[95% CI]	–	[0.9998, 1.0000]	[0.117, 1.176]	[0.863, 1.073]	[0.954, 1.008]	[0.967, 1.011]	[0.960, 1.011]	[0.709, 1.009]	[0.948, 1.001]
p-value		0.282	0.101	0.529	0.217	0.408	0.297	0.077	0.066
Odds ratio, Ct value	0.874	0.875	0.878	0.879	0.873	0.874	0.874	0.853	0.852
[95% CI]	[0.764, 1.005]	[0.765, 1.006]	[0.769, 1.009]	[0.767, 1.012]	[0.764, 1.004]	[0.765, 1.005]	[0.765, 1.005]	[0.741, 0.986]	[0.740, 0.985]
p-value	0.052	0.054	0.060	0.065	0.050	0.053	0.051	0.028*	0.027*
**AIC**	**189.2**	**189.9**	**188.4**	**190.8**	**189.6**	**190.5**	**190.1**	**187.8**	**187.6**

**Abbreviations:** AIC, Akaice Information Criterion; CI, confidence interval; Ct, cycle threshold values; *** p < 0.001, ** p < 0.01, * p < 0.05.

Odds ratios represent the odds ratio of PCR positivity associated with a one-unit increase in the predictor and are presented for various benznidazole exposure metrics and baseline Ct values. Results are shown both including and excluding the placebo group and a subject who took only 4 doses of benznidazole.

^a^based on beta binomial regression and an estimated overdispersion coefficient;

^b^based on binomial regression and a dispersion factor of 1;

^c^according to the historically accepted therapeutic range: 3–6 mg/L in plasma (2.5-5 mg/L in DBS);

^d^total days of benznidazole treatment (actual number of days benznidazole was taken);

^e^a week is counted if at least one benznidazole dose per week was taken;

^f^total duration of benznidazole treatment irrespective of intermittent drug interruption.

In the full mITT population (n = 201, including placebo), a significant relationship was observed between benznidazole exposure and the odds of qPCR positivity (p < 0.001) for all investigated PK/PD index parameters. The strength of this association was highly sensitive to the transformation of the exposure variable, as shown with the example of AUC_∞_. When using time above the target concentration as the predictor, parameter estimates and goodness-of-fit were also highly sensitive to the choice of the target concentration (S5 Fig), which remains unknown in vivo.

The exposure-response relationship was primarily driven by the placebo group (i.e., there was a clear difference in parasitological outcomes between no drug and all active regimens). After excluding placebo and the one patient who took only four doses of benznidazole, the exposure-response relationship was no longer significant (p > 0.05). While longer durations of treatment (whether expressed in weeks or total duration) suggested a marginal, non-significant trend towards lower probabilities of qPCR positivity, no clear PK/PD driver of qPCR positivity was identified in the remaining benznidazole treatment groups (n = 170 subjects). Goodness-of-fit diagnostics showed similar AIC values across different exposure parameters, with no improvement in model fit compared to a baseline model that included only Ct values. To aid interpretation of model estimates, probabilities of having at least one qPCR-positive follow-up for patients with multiple visits were calculated (S6 Fig). This transformation accounts for increased chances of detecting *T. cruzi* parasites at least once as the number of follow-up visits increases.

A trend towards a higher probability of post-treatment qPCR positivity was observed with lower Ct values (indicating higher baseline parasite loads). This trend remained significant or borderline significant even after excluding the placebo group and the influential subject, with an odds ratio consistently between 0.85 and 0.88 across models per unit increase in Ct (Table 3). Adjustment for combination therapy with fosravuconazole in this subgroup analysis did not show a significant effect on qPCR positivity.

Similar results were obtained in the refined mITT population (n = 186), excluding all subjects not meeting the PK/PD reliability criteria (S3 Table), though with reduced statistical power.

## Discussion

Current efforts to improve the anti-parasitic treatment of chronic *T. cruzi* infection have focused on reducing benznidazole exposure to enhance tolerability while maintaining efficacy [29,30,32,35,37–39,57–60]. In our study, we characterized the population pharmacokinetic properties of benznidazole, and we explored the relationship between benznidazole exposure and parasitological recrudescence using data from BENDITA. To our knowledge, this represents the largest population PK and PK/PD analysis of benznidazole in CD patients to date.

### Pharmacokinetic properties of benznidazole

Benznidazole concentration-time profiles were well described by a transit-compartment absorption model, followed by a one-compartment disposition model with linear elimination. The transit-compartment model provided a better fit and a more physiological representation of benznidazole’s absorption dynamics compared to the earlier first-order absorption models [26,27,29,57]. We observed substantial IIV in mean transit time (~62%), consistent with previous reports of high variability in absorption rates [48]. This variability is likely related to factors such as gastrointestinal pH, gastric emptying times, and food intake, which are potentially relevant because of benznidazole’s poor water solubility. The median T_MAX_ in our study (approximately 2 hours) was slightly shorter than the 3–4 hours reported in earlier studies [27,29,48], possibly due to differences in tablet formulations (Abarax vs. Radanil) and food states.

The one-compartment disposition model found in this study aligns with previous population pharmacokinetic analyses in both adult [29] and pediatric [30,31] CD patients. Our parameter estimates—V/F of 31.6 L, CL/F of 1.3 L/h—are in good agreement with earlier studies [48,57] and suggest moderate distribution into body tissues. Wiens et al. conducted a meta-analysis of the single-dose pharmacokinetics of benznidazole in adults and reported similar values (V/F ~ 39 L, CL ~ 2 L/h). Our elimination half-life estimates (~ 17h for monotherapy and ~ 14h for combination therapy) are somewhat longer than the than the 13.3 hours reported by Wiens et al. [48] and the 12–15 hours observed by Raaflaub et al. [26]. Soy et al. reported an even longer half-life (36 hours), potentially explained by different characteristics in the study population and food effects.

Benznidazole is extensively metabolized, most likely in the liver and possibly involving cytochrome P450 enzymes, though the specific metabolic pathway remains largely unknown [43,61,62]. Coadministration of fosravuconazole significantly increased benznidazole clearance, resulting in decreased benznidazole exposure. However, the effect was small (< 20%) and likely not clinically relevant. This is consistent with previous findings from a drug-drug interaction study in healthy volunteers [61].

Men exhibited lower relative bioavailability of benznidazole compared to women. In contrast, Molina et al. reported a higher volume of distribution (V/F) in men compared to women [57], and. Soy et al. did not find significant covariate effects [29]. The sample sizes in these studies were much smaller. In our study, both covariate effects—fosravuconazole coadministration on elimination clearance and the difference in relative bioavailability between sexes - were likely not relevant clinically. Nonetheless, these effects were included in the final model to describe the pharmacokinetic properties of benznidazole fully.

### PK/PD relationship for benznidazole

Evaluating antiparasitic drug efficacy in CD is remarkably challenging as there are no validated early biomarkers for clinical efficacy, and confirmation of parasitological cure is technically difficult as blood trypomastigote densities are often near or below the limit of detection [43]. In our study, we used the proportion of positive PCR results (qPCR positivity) as an endpoint for the PK/PD analysis. By modeling the outcome as a binomial random variable, we maximized information from multiple PCR samples and avoided bias against shorter treatment arms, which have more follow-up visits post-EOT and, therefore, higher chances of positive PCR results.

In the placebo arm, nearly all patients (29/30) did not clear their parasites, while the majority of patients in all benznidazole treatment arms had undetectable parasitaemia over one year follow-up. The clear difference in qPCR positivity between those receiving no treatment and those receiving any benznidazole highlights its potent anti-*T. cruzi* activity.

There was a non-significant trend suggesting higher post-treatment PCR positivity with the 2-week regimen compared to longer treatment courses, which has been examined in a recent probabilistic meta-analysis of these data [45]. However, the BENDITA trial was not powered to make definitive comparisons between active treatment regimens [32], and the 95% CIs for “treatment failure” proportions overlapped. Moreover, our analysis included all available post-treatment qPCR data, whereas the original BENDITA study excluded selected follow-ups to standardize visit numbers, limiting direct comparability. After excluding the placebo group and one patient who discontinued after only four doses of benznidazole (taken over 8 days), no clear pharmacokinetic predictor of treatment response was identified. It is an important finding that the once-weekly regimen, which provided the lowest total dose (AUC_∞_ 8-fold lower compared to the standard regimen), showed no apparent difference in efficacy compared to the 4-week or 8-week continuous treatment regimens. This suggests that time, in addition to benznidazole exposure, may be an important determinant of the therapeutic response. Together, these findings suggest that the standard 8-week regimen may be excessive.

Similar to the BENDITA trial, the MULTIBENZ trial explored shorter benznidazole treatment durations in the treatment of CD and found that a 15-day regimen of a higher dose (400mg instead of 300mg/day) was not inferior to the standard 60-day regimen, with fewer discontinuations resulting from adverse effects [35]. As in this study, the MULTIBENZ study did not observe a correlation between benznidazole concentrations and treatment outcomes. Together, these trials provide support for shorter or lower-dose regimens. However, the higher rate of qPCR positivity among patients with poor adherence emphasizes the importance of adhering to prescribed regimens, particularly for very short treatment durations.

In addition to exploring shorter treatment durations, lower doses have also been proposed. Soy et al. demonstrated through modeling and simulation that a reduced dose of 2.5 mg/kg/day could maintain plasma concentration within the historically accepted therapeutic range (3–6 mg/L) [29], supporting the idea that lower doses might be sufficient [30,63]. We confirmed that target concentrations (3–6 mg/L) could be achieved with lower doses, although results were sensitive to the threshold used. The historically accepted target range was extrapolated from tissue culture studies with limited methodological details [28], and while cited widely, the in vivo target concentration remains unknown. Furthermore, variability in drug susceptibility across different *T. cruzi* strains and regional differences in efficacy, as seen in MULTIBENZ, compromise generalisations over optimum treatment [64,65].

It has also been suggested that maintaining benznidazole levels above a target concentration is not essential, and that C_MAX_ is the primary driver of efficacy [40,41,66]. The existence of spontaneous dormancy [67] or transient non-replicative states [68–70] remains a matter of debate and has been hypothesized to contribute to treatment failure due to their reduced susceptibility to drug-induced toxicity. Intermittent dosing of sufficiently high doses over an extended period could potentially address this challenge, while reducing total drug exposure and potentially lowering toxicity.

Given the complexity of CD, and the possibility of a transient non-replicative state, it is also plausible that more than one predictor of benznidazole efficacy - such as a combination of treatment duration and C_MAX_ or AUC_∞_ - is needed to adequately capture the relationship between exposure and treatment efficacy. Several pre-clinical studies have emphasized the critical role of both benznidazole dose and treatment duration in achieving parasitological cure in *T. cruzi* infected mice [41,71–74]. However, high collinearity between PK/PD index parameters hampers the identification of the primary driver of efficacy or combination of drivers. Improved study designs, such as dose fractionation studies, have been suggested to help disentangle these factors and better understand the determinants of benznidazole efficacy [75].

The BENDITA trial assessed intermittent dosing of benznidazole with fosravuconazole co-administration, finding similar efficacy to the standard regimen. However, the design of the trial did not allow discrimination of the effects of fosravuconazole from those of intermittent treatment.

### Strengths and limitations

A strength of our study was the application of nonlinear mixed-effects modeling to characterize the pharmacokinetic properties of benznidazole, which enabled a pooled analysis across treatment arms, effective handling of sparse PK data, and facilitated the identification of covariate effects. The modeling also enabled the identification of patients with outlying PK samples, allowing for the recognition of potential issues such as non-adherence or potential discrepancies in treatment allocation. Despite some inconsistencies in PK data, sensitivity analyses confirmed the robustness of our findings.

The study has several limitations. The BENDITA trial was not powered to differentiate between active treatment arms or to identify the primary driver of benznidazole efficacy, nor could it separate the effects of the azole drug from the different treatment regimens. PCR, while widely used in clinical trials, including BENDITA, has limitations in estimating absolute cure rates due to the low density of *T. cruzi* in blood near the detection limit and challenges in interpreting qPCR results from the few samples collected. New biomarkers of clinical efficacy and disease progression are urgently needed. Additionally, the study’s one-year follow-up may not be sufficient to assess long-term parasitological outcomes. A placebo arm was included to enable a robust comparison of the antiparasitic activity of benznidazole and new treatment regimens. The inclusion of a placebo arm may be debated in light of earlier observational studies suggesting long-term benefits of benznidazole in CD patients without cardiac involvement [10,11]. However, due to the chronic nature of CD and its slow progression, there is no evidence of significant short-term risk within the trial evaluation period. Moreover, all participants were offered rescue treatment after study completion. Since the study was conducted in Bolivia, the findings may not be generalizable to other regions with different *T. cruzi* strains [35]. Furthermore, the in vitro IC_90_ used as a reference in this study was measured against the amastigote form of *T. cruzi* (Tulahuen strain), whereas qPCR detects circulating trypomastigotes in blood. The PK/PD index parameter T > IC_90_ is highly sensitive to the choice of the target concentration, as demonstrated by comparisons with the often-cited range (3–6 mg/L). The in vivo target concentration, however, remains unknown. Lastly, exposure correlates of toxicity and adverse events have not yet been analyzed separately; this will be addressed in future work.

## Conclusions

This study successfully described benznidazole’s population pharmacokinetics in adult patients with chronic CD, showing that fosravuconazole does not have a clinically relevant impact on benznidazole’s pharmacokinetics. Our analysis suggests that the standard 8-week dosing regimen may be excessive, as both weekly dosing for 8 weeks or daily dosing for 4 weeks appeared similarly effective. In contrast, there is an indication that the 2-week regimen may be less effective. No clear pharmacokinetic predictor of treatment failure was identified among those receiving benznidazole treatment. Future well-designed trials, ideally employing factorial randomization and complementary endpoints to qPCR, are needed to robustly evaluate the optimal balance of dose, duration, and frequency and to identify the most well-tolerated regimen that achieves maximal therapeutic efficacy.

## Supporting information

S1 Text Outlier analysis. (DOCX)

S2 Text Determination of in vitro antitrypanosomal activity. (DOCX)

S1 Fig Flow chart of excluded patients. (DOCX)

S2 Fig Distribution of benznidazole exposure variables across treatment arms. (DOCX)

S3 Fig Simulated pharmacokinetic profiles, by sex. (DOCX)

S4 Fig Number of follow-up visits. (DOCX)

S5 Fig Predicted probabilities of qPCR positivity for a single follow-up visit using different predictors. (DOCX)

S6 Fig Predicted probabilities of at least one follow-up visit being qPCR positive. (DOCX)

S1 Table Secondary pharmacokinetic parameter estimates. (DOCX)

S2 Table Correlation matrix of plasma variables for benznidazole DBS concentrations. (DOCX)

S3 Table Regression model diagnostics (sensitivity analyses). (DOCX)
