# Supplementary material for: Population pharmacokinetic-pharmacodynamic analysis of benznidazole monotherapy and combination therapy with fosravuconazole in chronic Chagas disease (BENDITA)
Source: PLoS Negl Trop Dis. 2025 Sep 22;19(9):e0013522. doi: 10.1371/journal.pntd.0013522 (PMC12510642; doi:10.1371/journal.pntd.0013522)
Supplement: S1 Text — (DOCX) [file pntd.0013522.s001.docx]

**S1 Text. Outlier analysis**

**S1.1 Initial exploratory analysis and methodology**

During the initial exploration of benznidazole pharmacokinetic (PK) profiles (**Fig. A** ), inconsistencies were observed in several subjects. Notably, 26 subjects showed benznidazole levels above the LLOQ at ≥120h time after dose (TAD), which represents approximately 10 half-lives. These observations were classified as 'TAD outliers'. Additionally, inconsistencies were noted where benznidazole levels fell below the LLOQ at unexpected times, such as around T_MAX_, classifying these as 'BQL outliers'.

**
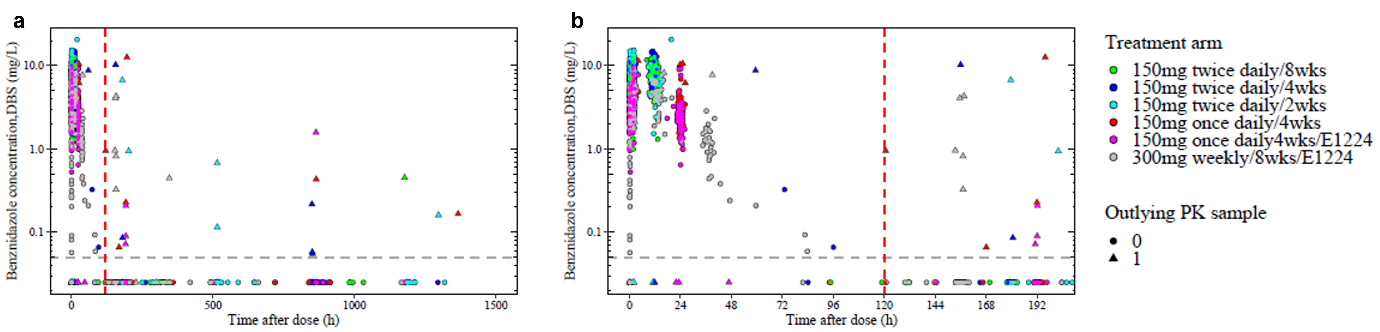
**

**Fig A.** Benznidazole concentrations in blood vs. time after dose (TAD) in patients allocated to active treatment arms (n = 180) in the BENDITA study. **A)** shows the entire time scale, **B)** zooms in on the first 200 h of time after last dose. The red vertical line presents the 120h TAD interval (~ 10 half -lives); the grey horizontal line presents the LLOQ. Shape by outlying PK sample (circle: no outlier, triangle: outlier).

These findings prompted a systematic outlier analysis. The methodology used to identify outlying PK samples or profiles and additional inconsistencies in drug exposure is summarized in **Figure B.**

A preliminary population PK model for benznidazole was developed, including all subjects receiving active treatment (n=180), with PK data censored at 120 hours TAD. Concentrations below the LLOQ were excluded (M1 BQL method) [1]. This preliminary PK model was applied to identify PK outliers through simulation-based diagnostics (simeval tool in PsN 5.2.6) [2,3], and influential outliers were detected through case deletion diagnostics (qa tool in PsN) [4].


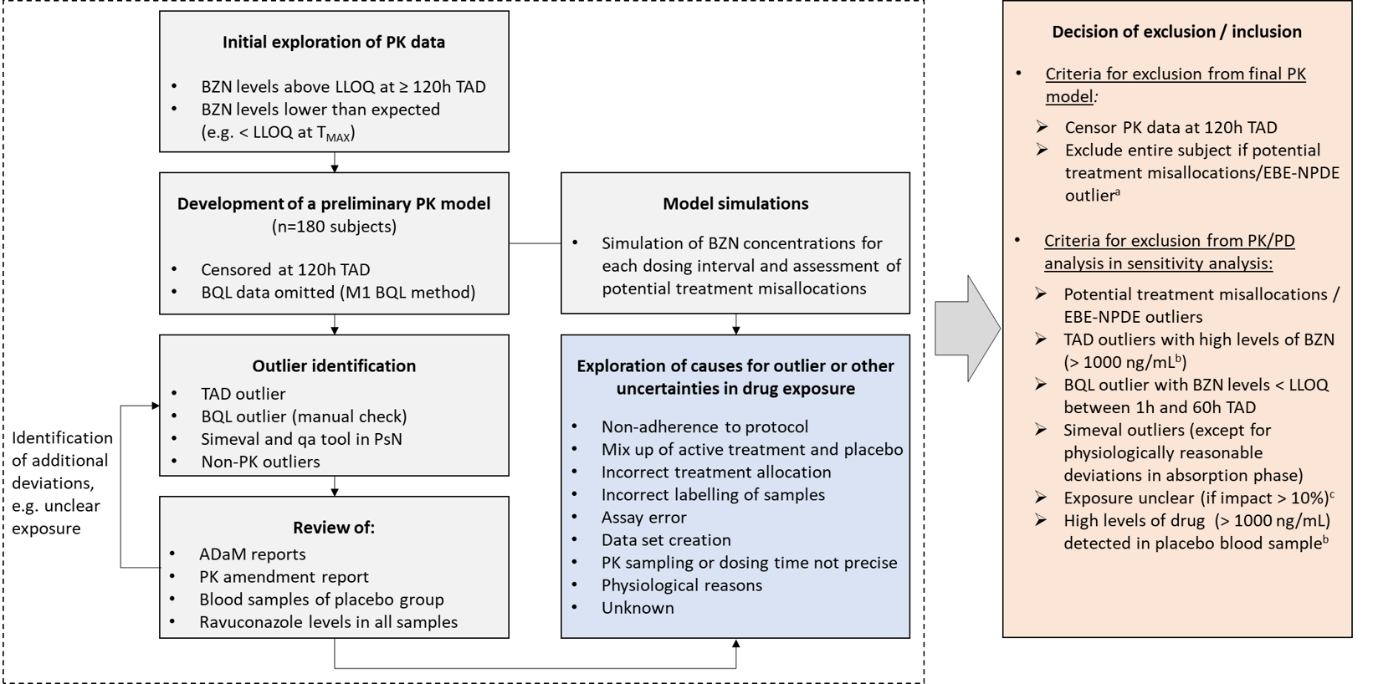


**Fig B**. - Methodology for identification of PK outliers and other uncertainties in drug exposure. **Abbreviations:** BZN, benznidazole; LLOQ, lower limit of quantification; TAD, time after dose; T_MAX_, time to maximum concentration; BQL, below quantification level; qa, quality assurance; PK, pharmacokinetic; EBE-NPDE, empirical bayesian estimates- normalized prediction distribution errors. ^a^ Outlier exhibiting markedly different PK parameter estimates compared to the population mean. ^b^ A threshold of 1000 ng/mL was set, corresponding to approximately 5% of C_MAX_. ^c^ Subjects for which the exposure was unclear were excluded if the impact would be > 10% on BZN exposure.

Additionally, data from several sources were reviewed to ensure data integrity, including: ADaM datasets (e.g., for drug administration, concentrations, dosing deviations, drug accountability, adverse events) and the bioanalytical report. After unblinding, unexpected drug concentrations in placebo and baseline samples prompted a re-analysis of PK samples from 2017 due to quality issues, and selected 2018 samples with allocation inconsistencies. Placebo samples were also checked for drug levels.

Subjects identified as PK outliers or with other exposure uncertainties were classified by detection method. Potential causes were then investigated by reviewing all available data, and a decision matrix was applied to guide inclusion or exclusion from the final PK and PK/PD analyses.

References:

1. Beal, S.L., Ways to fit a PK model with some data below the quantification limit. J Pharmacokinet Pharmacodyn, 2001. 28(5): p. 481-504.

2. SIMEVAL user guide, PsN 5.3.1, Revised 2019-04-03.

3. Largajolli, A., S. Jönsson, and K. M.O., The OFVPPC: A simulation objective function based diagnostic, in PAGE. 2014: Alicante, Spain

4. QA user guide, PsN 5.3.1, Revised 2019-10-15.

**S1.2 Subjects with uncertain exposure (by diagnostics)**

Among the 210 participants in the BENDITA study, 52 subjects (25%) exhibited unexpected drug levels or other drug exposure-related uncertainties (**Figure C**)**.**

Within the active treatment arm (n=180), 39 subjects (22%) were identified as PK outliers, characterized by pronounced discrepancies between observed and predicted benznidazole concentrations (mostly TAD outliers). An additional 8 subjects in the active treatment arm were not PK outliers, but presented with other uncertainties, such as ravuconazole detection in blood samples from a benznidazole monotherapy arm or ambiguous benznidazole exposure (possibly due to mix-ups between placebo and active treatment, missed doses, labelling or data set creation errors). In the placebo group, 5 subjects had detectable levels of benznidazole or ravuconazole in blood.

**
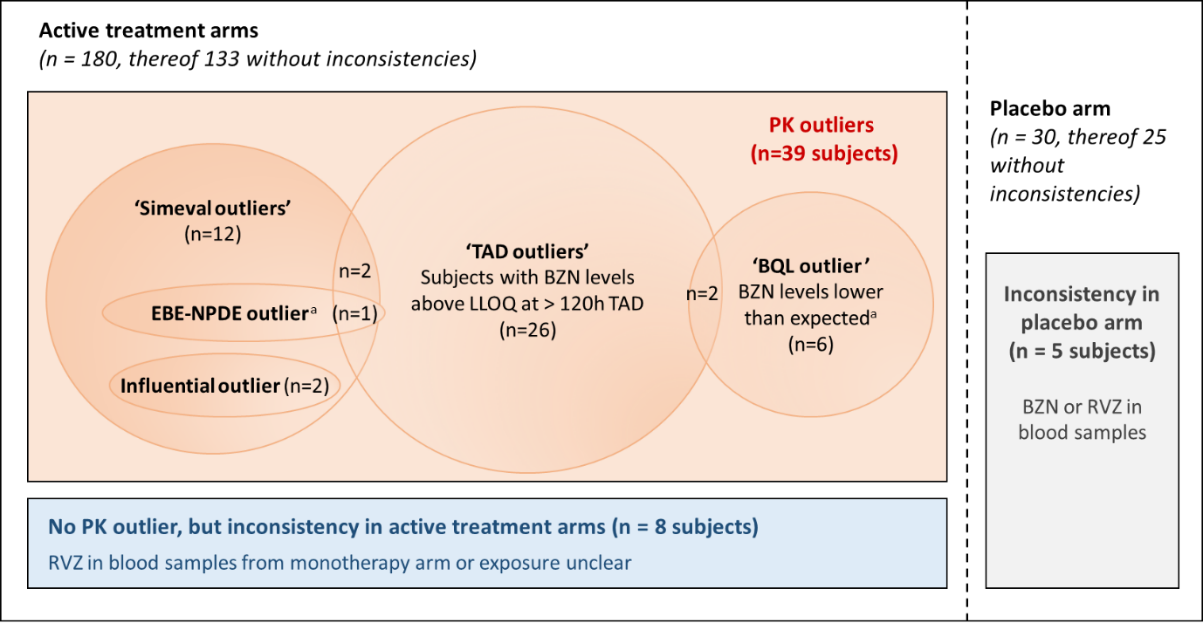
**

**Fig C.** Classification of the 52 subjects identified with deviations in drug concentration levels or uncertainties related to drug exposure. **Abbreviations:** BZN, benznidazole; RVZ, ravuconazole; TAD, time after dose; LLOQ, lower limit of quantification; EBE-NPDE, empirical Bayesian estimates- normalized prediction distribution errors.

**S1.3 Subjects with uncertain exposure (by potential cause)**

The specific reasons for problematic PK data remained uncertain for most subjects, with multiple factors potentially contributing. **Table A** summarizes the most likely explanations.

Most PK outliers were attributed to potential bioanalytical assay issues, accounting for 22 subjects (10.5 % of the total study population). Many TAD outliers and inconsistencies in the placebo arm exhibited low BZN concentrations (<1000 ng/mL). Some PK outliers were detected solely in the re-analyzed samples, further supporting the possibility of assay errors.

Other sources of inconsistency included a possible mix-up between active treatment and placebo in the once daily or weekly treatment arms. The outlying PK samples for two patients were identified and resolved by addressing a documented mix-up. One outlying PK sample was traced back to a data set creation error, though other issues remained.

Finally, four patients had PK profiles that were inconsistent with their assigned treatment regimen and showed stronger alignment with an alternative treatment arm, suggesting the possibility of incorrect treatment allocation (**Figure D**). For one additional patient, the cause of the outlying PK samples was flagged as unknown, but treatment misallocation could not be entirely ruled out.

**Table A.** Summary of the potential causes of inconsistencies for each subject (n=52), with only the most likely cause/s shown.

| Most likely reason for outlying PK sample or other inconsistency | Definition | N subjects  (% out of 52) |
| --- | --- | --- |
| Assay error | Issues with the bioanalytical assay, e.g. changes to the technique over time, carry-over effects, drug instability, sample contamination, or responses from concomitant drugs or metabolites, compromised sample integrity due to issues with sample processing, storage or transportation. If BZN concentrations were low compared to typical values (< 1000 ng/mL for all TAD outliers), the outlier was flagged as a potential assay error. | 22 (42.3%) |
| Mix-up active treatment and placebo | Uncertainty if the patient received the active drug or placebo, particularly across the once daily or weekly treatment arms, due to the nature of a double -blind, placebo-controlled study design and incomprehensive ADEX report (whether active treatment or placebo was taken). | 7 (13.5%) |
| Non-adherence to the protocol | Failure of patient to comply with the planned dosing regimen, either by not taking the drug as instructed or by providing inaccurate reports of their medication intake. | 4 (7.7%) |
| Incorrect treatment allocation | Observed PK profile does not match the assigned treatment arm but aligns more closely with another arm. | 4 (7.7%) |
| Physiological reasons | The observed deviation may be genuine and attributable to physiological factors, such as autoinduction or variations in absorption rate. | 5 (9.6%) |
| Incorrect labelling of samples | Mislabelling of samples. | 3 (5.8%) |
| Data set creation | Inaccurate data entry (e.g. typing error, transcription error). | 1 (1.9%) |
| Unknown (one subject for which incorrect treatment allocation cannot be excluded) | Used when the cause is uncertain and does not fit into any of the other defined categories (e.g. assay error, mix-up, non-adherence). Other possible reasons include e.g. incorrect blister content: either placebo instead of active or vice versa). | 2 (3.8%) |
| PK sampling or dosing time not precise | PK sampling time or dosing time recorded inaccurately. | - |
| Multiple potential causes of inconsistencies per subject: | | |
| Assay error or Mix-up |  | 1 (1.9%) |
| Assay error and Data set creation |  | 1 (1.9%) |
| Non-adherence or Mix-up; and Assay error |  | 1 (1.9%) |
| Assay error and Non-adherence |  | 1 (1.9%) |

| **Patient 1061**  allocated to 150mg BZN/QD/4weeks/ E1224) is a BQL outlier (in week 3 and 4), raising the possibility of treatment misallocation to the weekly BZN treatment. | 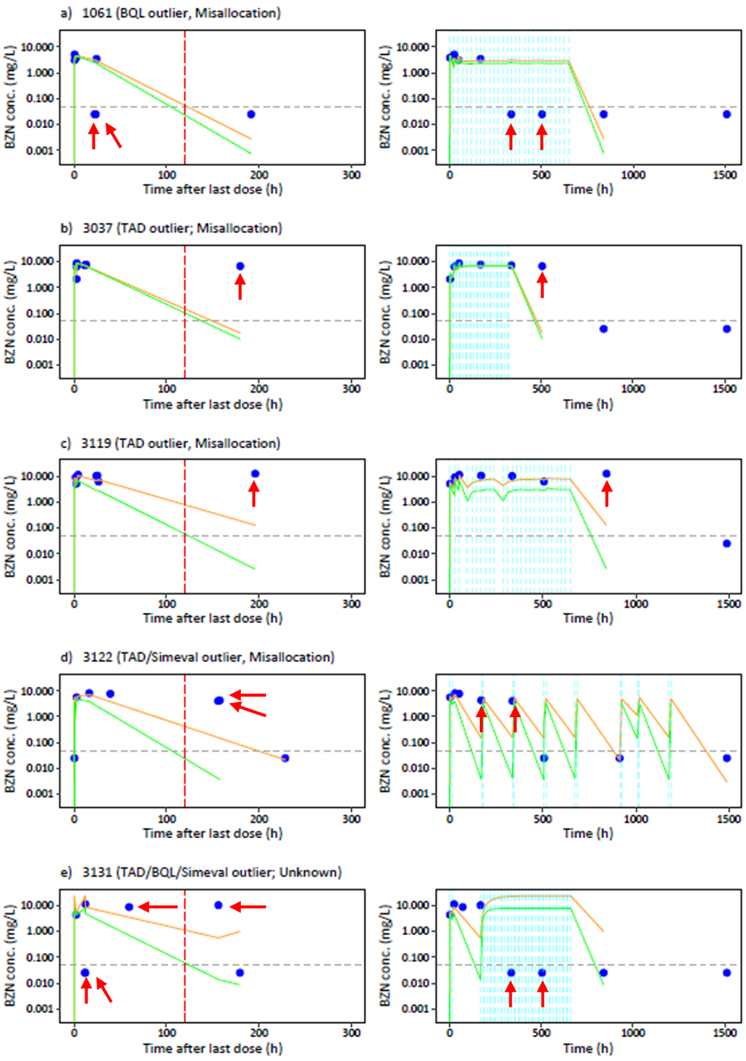 |
| --- | --- |
| **Patient 3037**  allocated to 150mg BZN/BID/ 2weeks) is a TAD outlier with high benznidazole levels (>> 1000 ng/mL) in week 4, raising the possibility of treatment misallocation to 4 weeks treatment. | 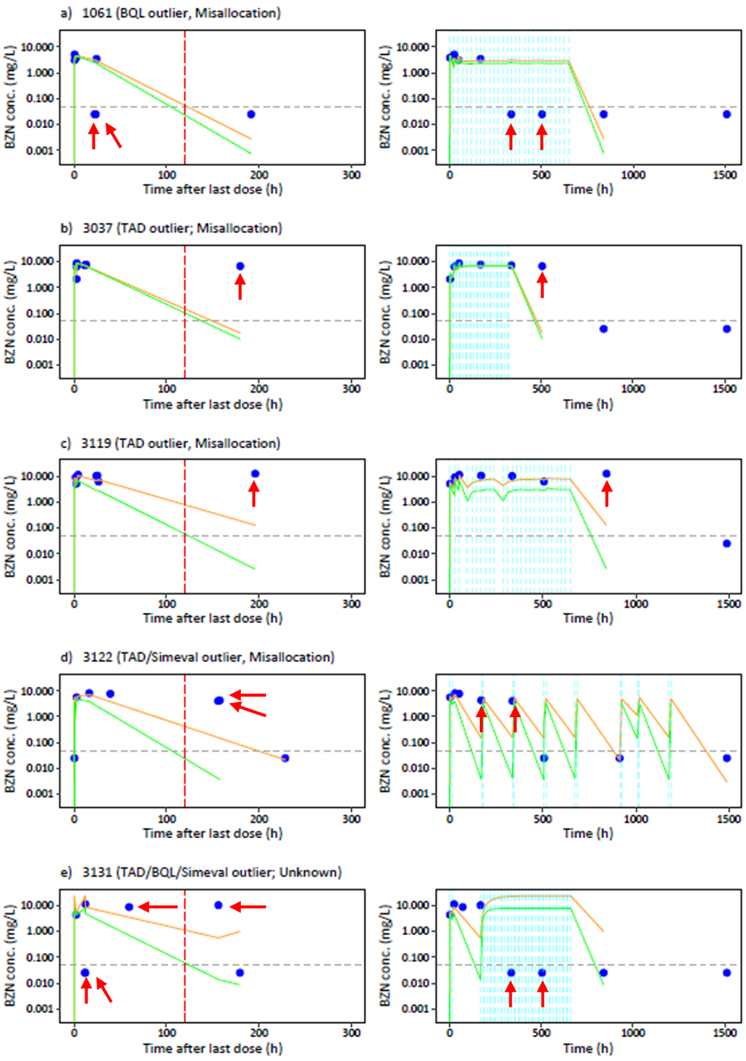 |
| **Patient 3119**  allocated to 150mg BZN/QD/4 weeks is a TAD outliers with high benznidazole levels (>> 1000 ng/mL) in week 6, raising the possibility of treatment misallocation to 8 weeks treatment. | 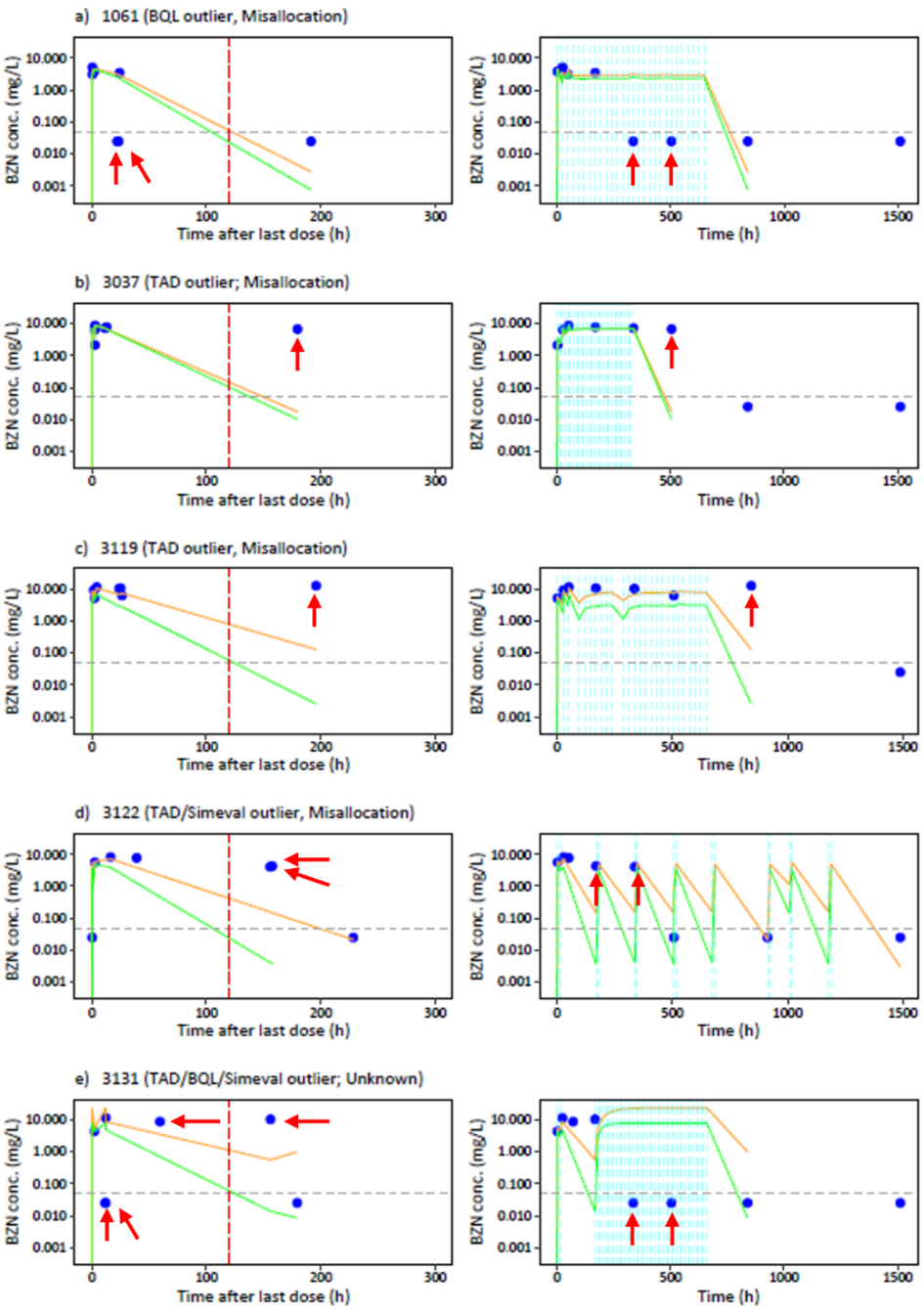 |
| **Patient 3122**  allocated to 300mg/weekly/ 8w/E1224 is a TAD and Simeval outlier. The patient was allocated to a combination treatment arm, but no ravuconazole was detected in blood at any time, raising the possibility of treatment misallocation (to 2 weeks BZN monotherapy). | 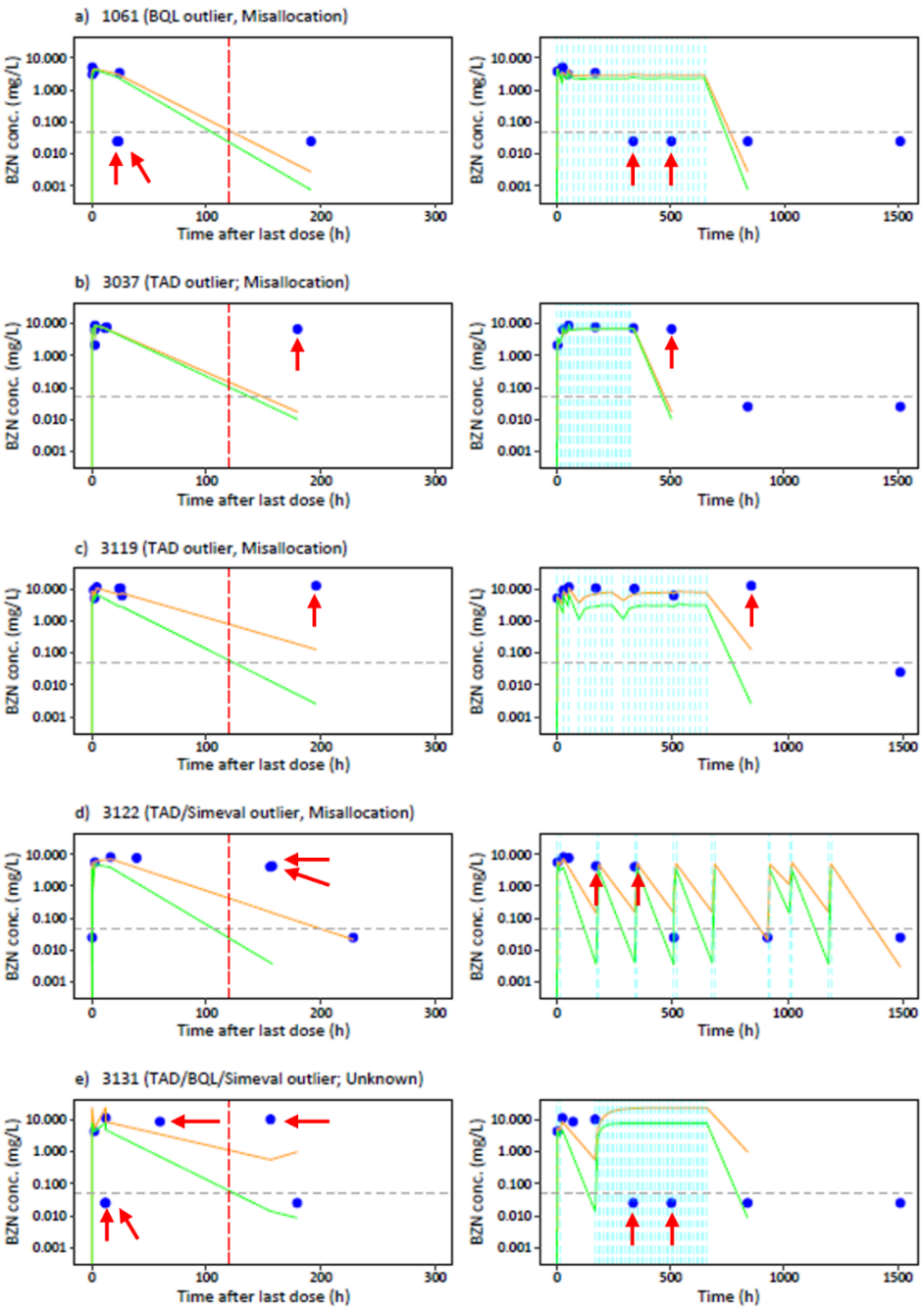 |
| **Patient 3131**  allocated to 150mg BZN/ BID/ 4weeks) is the only EBE-NPDE outlier and also a TAD and BQL outlier. The exact reason for these outliers remains elusive, the possibility of treatment misallocation cannot be ruled out. | 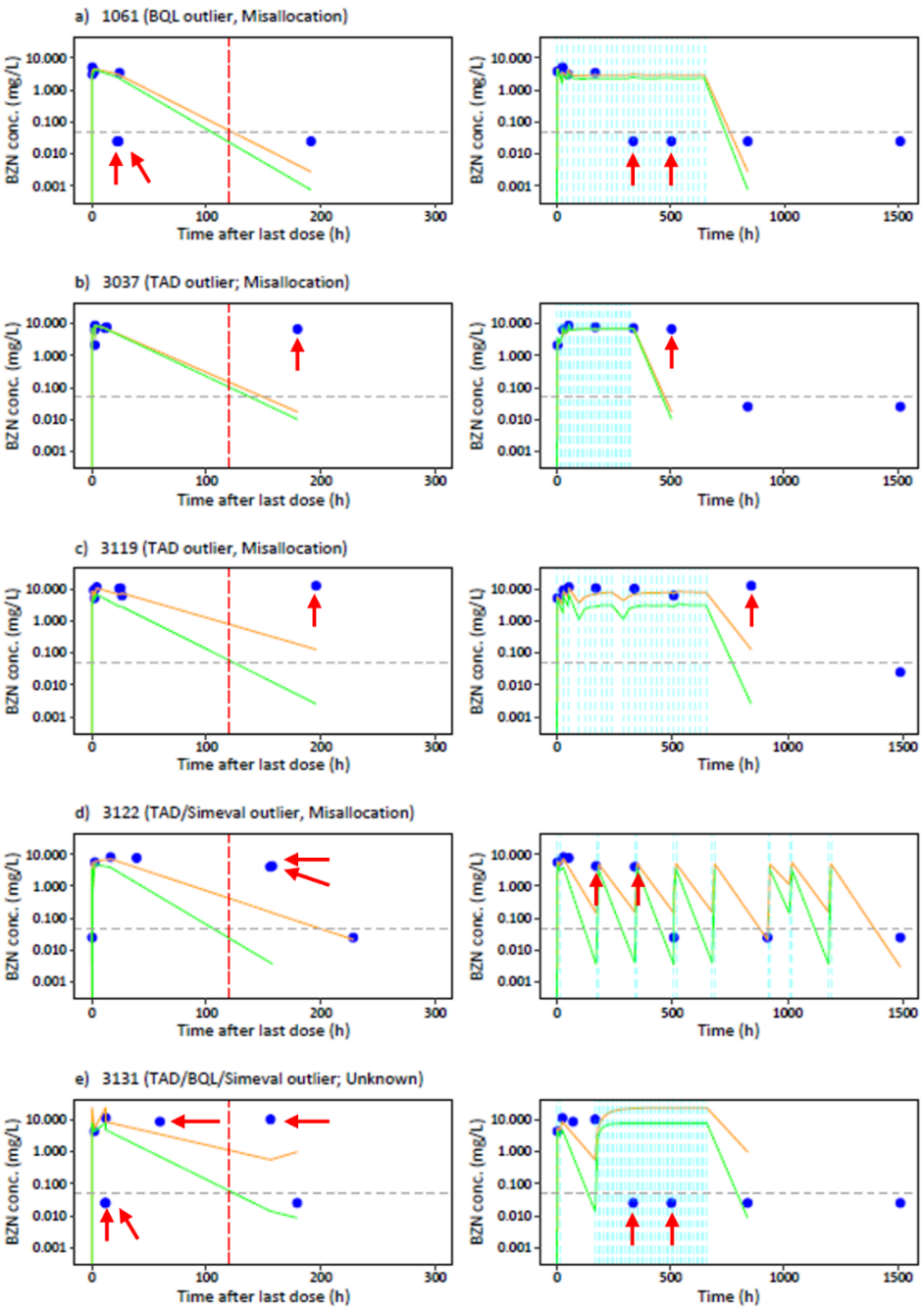 |

**Fig D.** PK profiles for patients with significant PK outliers, potentially indicative of treatment misallocation. **Left panel:** Benznidazole concentrations versus time after the last dose (zoom into the first 300h TAD). The red vertical line presents the 120h TAD interval (approximately 10 half -lives); while the grey horizontal line presents the LLOQ. **Right panel:** Benznidazole concentrations versus time. Light-blue vertical lines indicate benznidazole dosing times as reported. Blue circles represent observed benznidazole levels in dry blood spots. Green and orange lines depict population predicted and individually predicted benznidazole concentrations, respectively, using the preliminary PopPK model. Red arrows indicate outlying PK samples. In brackets are outlier categories by diagnostics and the most likely cause.

**S1.4 Exclusion criteria and summary of excluded subjects**

Following the systematic assessment of PK outliers and inconsistencies in exposure, subjects were excluded from the PK and/or PK/PD analyses based on predefined criteria (see **Info Box**). Whenever the reason for an outlying PK sample could be identified, the issue was addressed, and the subject was included in all PK and PK/PD analyses, provided no other inconsistencies were found.

| **Info Box. Exclusion criteria (based on diagnostics)**  **Exclusion from the final PK model**   - PK data censored at 120h TAD 🡪 Exclude all outlying PK samples (not subjects) with benznidazole levels above the LLOQ at ≥120 h after the last dose. - Exclude subjects if PK profiles showed strong inconsistencies with the assigned regimen, raising the possibility of treatment misallocation.   **Exclusion from PK/PD analysis**   - Same criteria as for PK model, plus: - For sensitivity analysis, exclude entire subject with: |
| --- |
| - TAD outlier showing high levels of benznidazole (> 1000 ng/mL), unlikely an assay error - BQL outliers - Simeval outliers, except those with plausible physiological explanations - Unclear exposure potentially impacting benznidazole dose by > 10% (e.g., possible mix-ups of placebo and active treatment) - High drug levels (>1000 ng/mL) in placebo samples. |

- In total, **5 subjects** with potential treatment misallocation **were excluded from the final PK and PK/PD analyses**. PK data were also censored at 120h TAD.
- Stricter criteria were applied **for the PK/PD sensitivity analysis**, excluding **22 subjects with significant PK outliers or uncertain exposure, including cases where treatment misallocation could not be ruled out**.
- Notably, two subjects identified as influential by the qa tool were retained in the PK or PK/PD analysis datasets. Both showed reasonable PK profiles, with outlying PK samples that could be attributed to physiological variability in absorption kinetics (dOFV values after case deletion were relatively small: 5.0 and 3.9).

S1.5 Sensitivity analysis

To assess the robustness of the PK model, a sensitivity analysis was conducted by excluding the five patients with significant PK outliers, potentially indicative of treatment misallocations (censored data). The analysis showed minimal changes in population PK parameters, indicating the robustness of the PK model even in the presence of potential misallocations.

**Table B.** Parameter estimates of the final population PK model of benznidazole.

|  | **Population estimate (IIV, %CV) ^a^** | |
| --- | --- | --- |
| **Parameter** | **All subjects (n=180)** | **Excluding potential misallocations (n=175)** |
| Relative oral bioavailability, F | 1 *fixed* (10.4%) | 1 *fixed* (10.2%) |
| Mean transit time, MTT (h) | 0.757 (60.7%) | 0.753 (61.6%) |
| Apparent clearance, CL/F (L/h) | 1.28 (23%) | 1.30 (18.9%) |
| Apparent volume of distribution, V/F (L) | 31.8 (-) | 31.6 (-) |
| Co-administration of E1224 on CL/F (%) | 17.0 | 17.7 |
| Sex effect on F (reference: female) (%) | -12.6 | -12.9 |

Population estimates are given for an adult weighting 65 kg. ^a^ Population mean parameter estimates and IIV calculated by NONMEM.
