# Supplementary material for: Population pharmacokinetic-pharmacodynamic analysis of benznidazole monotherapy and combination therapy with fosravuconazole in chronic Chagas disease (BENDITA)
Source: PLoS Negl Trop Dis. 2025 Sep 22;19(9):e0013522. doi: 10.1371/journal.pntd.0013522 (PMC12510642; doi:10.1371/journal.pntd.0013522)
Supplement: S2 Text — (DOCX) [file pntd.0013522.s002.docx]

**S2 Text: Determination of in vitro antitrypanosomal activity**

In vitro activity of benznidazole against the amastigote form of *T. cruzi* (Tulahuen strain) in 3T3 host cells was determined using a high content, image-based analysis [1] .

The IC_90_ value for benznidazole in the assay medium (RPMI medium supplemented with 10% fetal calf serum) was determined to be ${IC}_{90,medium}$ = 17.6 µM. Since the binding of benznidazole in RPMI medium was minimal (<10%), an unbound fraction of $f_{unbound,medium}$  = 0.95 was assumed for scaling ${IC}_{90,medium}$ to ${IC}_{90,unbound}$, as follows:

${IC}_{90,unbound}= {IC}_{90,medium}\times f_{unbound,medium}$ . (1)

Protein binding for benznidazole in human plasma is moderate, with reported values of 44% and 60% protein-bound drug [2,3]. We assumed a mean value of 52% protein binding in human plasma. Accordingly, an unbound fraction of $f_{unbound,plasma}$ = 0.48 in human plasma was used to correct for plasma protein binding, as follows:

${IC}_{90,plasma}= \frac{{IC}_{90,unbound}}{f_{unbound,plasma}}$ . (2)

Finally, the ${IC}_{90,plasma}$ was scaled to the corresponding concentration in dry blood spots (DBS), based on a previously published regression analysis. Benznidazole concentrations collected as DBS and in plasma were highly correlated (r^2^ = 0.83), with DBS concentrations being approximately 20% lower than plasma concentrations . The regression line had a slope of 0.84, which was used for scaling. The derived total concentration of benznidazole in human DBS samples ( ${IC}_{90,DBS}$= 29.3 µM, i.e. 7.61 mg/L) was set as the target concentration in human.

References:

1. Sykes, M.L. and V.M. Avery, Development and application of a sensitive, phenotypic, high-throughput image-based assay to identify compound activity against Trypanosoma cruzi amastigotes. Int J Parasitol Drugs Drug Resist, 2015. 5(3): p. 215-28.

2. Raaflaub, J. and W.H. Ziegler, Single-dose pharmacokinetics of the trypanosomicide benznidazole in man. Arzneimittelforschung, 1979. 29(10): p. 1611-4.

3. Roberts, J.T., et al., A phase I study of the combination of benznidazole and CCNU in man. Int J Radiat Oncol Biol Phys, 1984. 10(9): p. 1745-8.

4. Galindo Bedor, D.C., et al., Dried Blood Spot Technique-Based Liquid Chromatography-Tandem Mass Spectrometry Method as a Simple Alternative for Benznidazole Pharmacokinetic Assessment. Antimicrob Agents Chemother, 2018. 62(12).
