# Supplementary material for: Population pharmacokinetic-pharmacodynamic analysis of benznidazole monotherapy and combination therapy with fosravuconazole in chronic Chagas disease (BENDITA)
Source: PLoS Negl Trop Dis. 2025 Sep 22;19(9):e0013522. doi: 10.1371/journal.pntd.0013522 (PMC12510642; doi:10.1371/journal.pntd.0013522)
Supplement: S1 Fig — (DOCX) [file pntd.0013522.s003.docx]

**
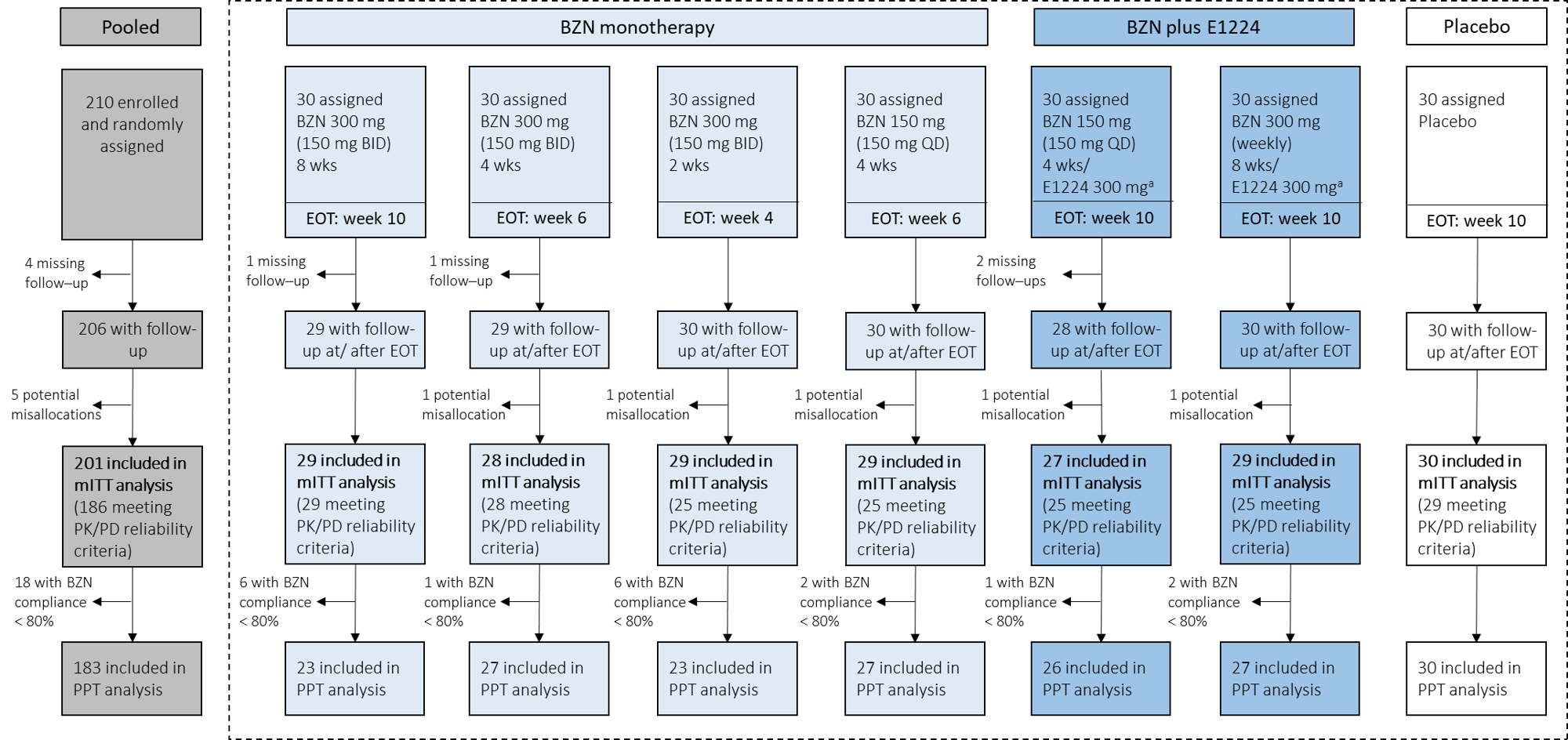
**

**S1 Fig.** Flow chart of excluded patients with respect to the pooled dataset (grey boxes) and by treatment arm. ^a^ Loading dose: 300 mg QD on days 1-3. Maintenance dose: 300 mg once weekly (week 2-8). In bold: modified ITT (mITT) population included in primary PK/PD. Subjects with significant PK outliers, potentially indicative of treatment misallocation, were excluded from all analyses.
