# Supplementary material for: Population pharmacokinetic-pharmacodynamic analysis of benznidazole monotherapy and combination therapy with fosravuconazole in chronic Chagas disease (BENDITA)
Source: PLoS Negl Trop Dis. 2025 Sep 22;19(9):e0013522. doi: 10.1371/journal.pntd.0013522 (PMC12510642; doi:10.1371/journal.pntd.0013522)
Supplement: S2 Fig — (DOCX) [file pntd.0013522.s004.docx]

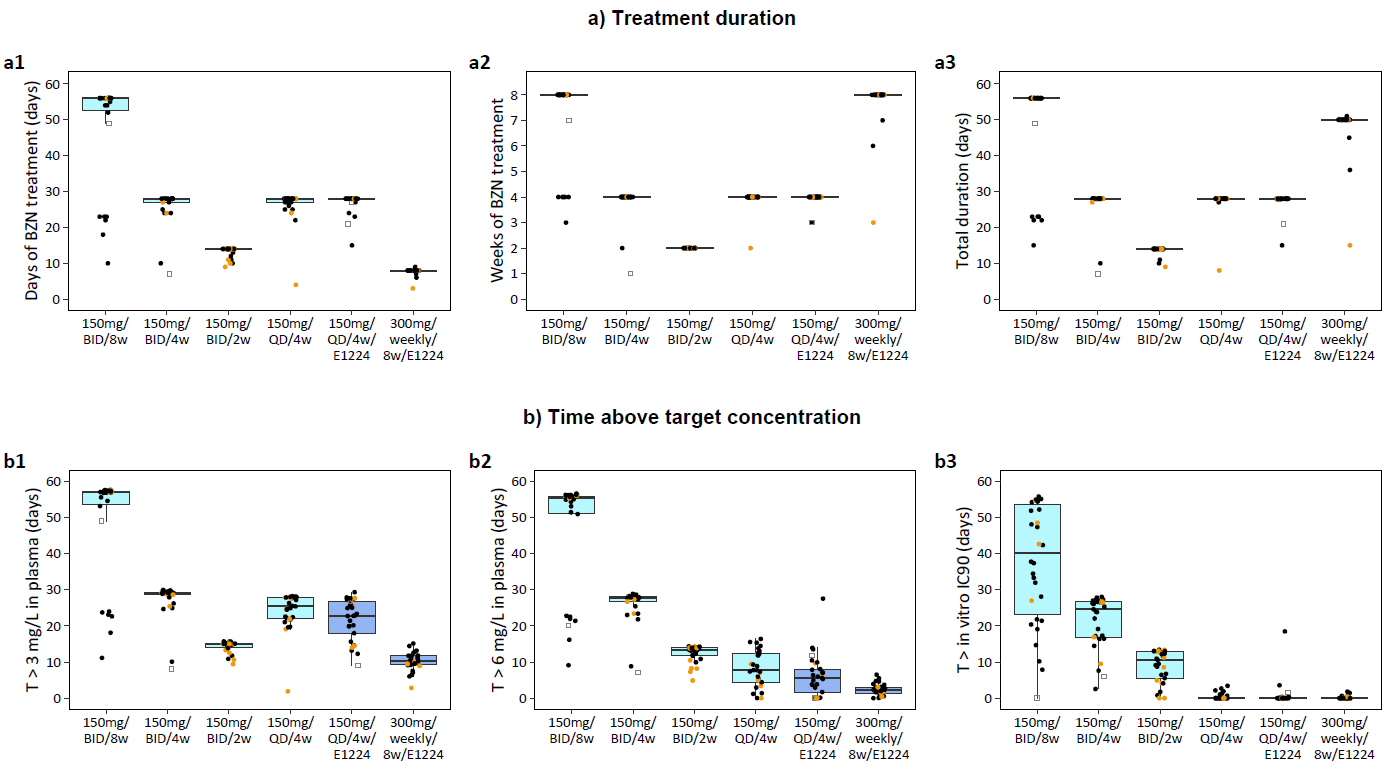


**S2 Fig.** Distribution of benznidazole exposure variables across treatment arms in the PK analysis population (n = 175).

**a)** Durations of benznidazole treatment using different definitions: **a1)** total days of benznidazole treatment (actual number of days benznidazole was taken), a**2)** weeks of treatment (a week is counted if at least one dose of benznidazole was taken), and **a3)** total treatment duration (regardless of any intermittent interruptions). **b)** Time above the target concentrations with different thresholds: **b1)** 3 mg/L in plasma, **b2)** 6 mg/L in plasma, and **b3)** scaled in vitro IC_90_;

Median (midline), interquartile range (IQR, box), and 1.5*IQR whiskers are shown. Boxplot colors indicate benznidazole monotherapy (light blue) or co-administration with E1224 (dark blue). Symbol shapes denote patient follow-up (FU) status post-treatment (filled circles: with FU, open squares: missing FU). Symbol colors represent treatment outcome (black: no treatment failure, orange: treatment failure). **Abbreviations:** BZN, benznidazole; QD, once daily; BID, twice daily; E1224, fosravuconazole
