# Supplementary material for: Population pharmacokinetic-pharmacodynamic analysis of benznidazole monotherapy and combination therapy with fosravuconazole in chronic Chagas disease (BENDITA)
Source: PLoS Negl Trop Dis. 2025 Sep 22;19(9):e0013522. doi: 10.1371/journal.pntd.0013522 (PMC12510642; doi:10.1371/journal.pntd.0013522)
Supplement: S3 Fig — (DOCX) [file pntd.0013522.s005.docx]

| 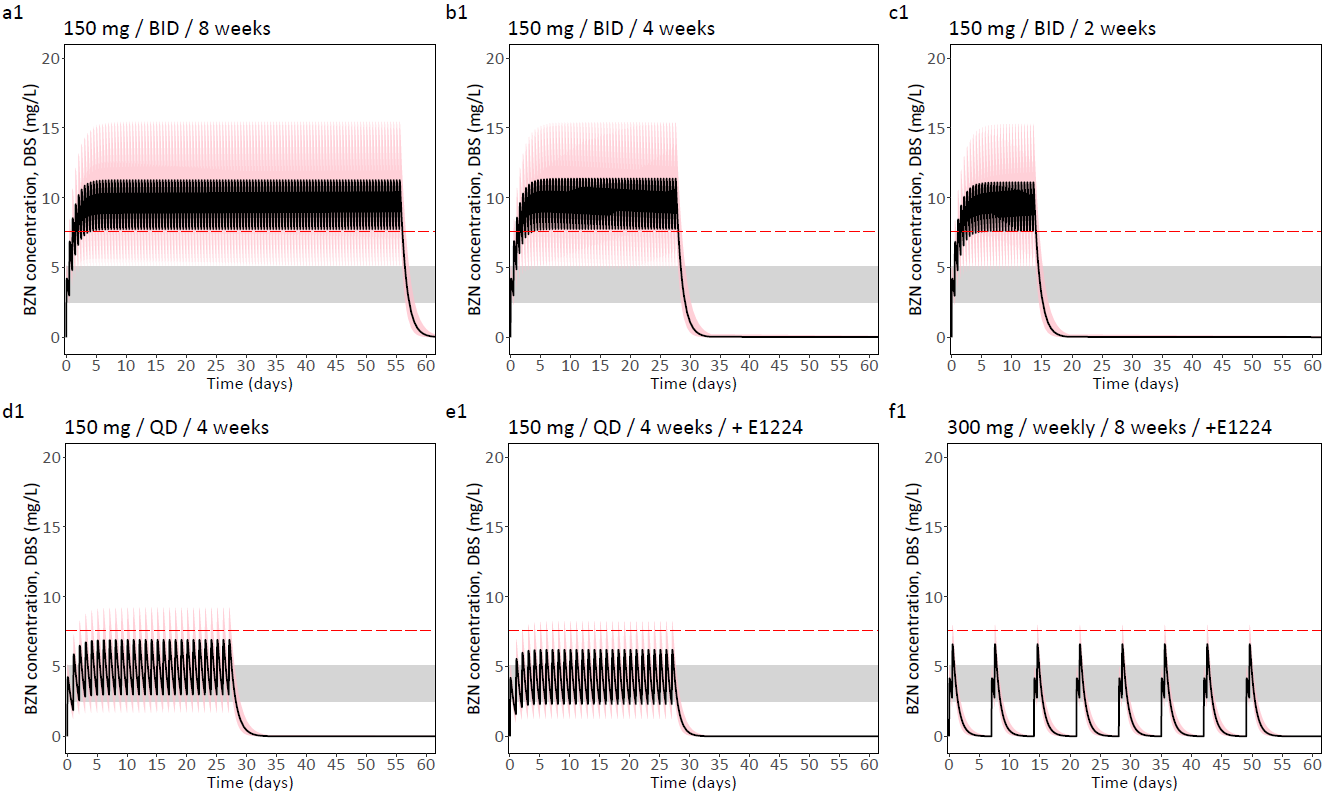 |
| --- |
| 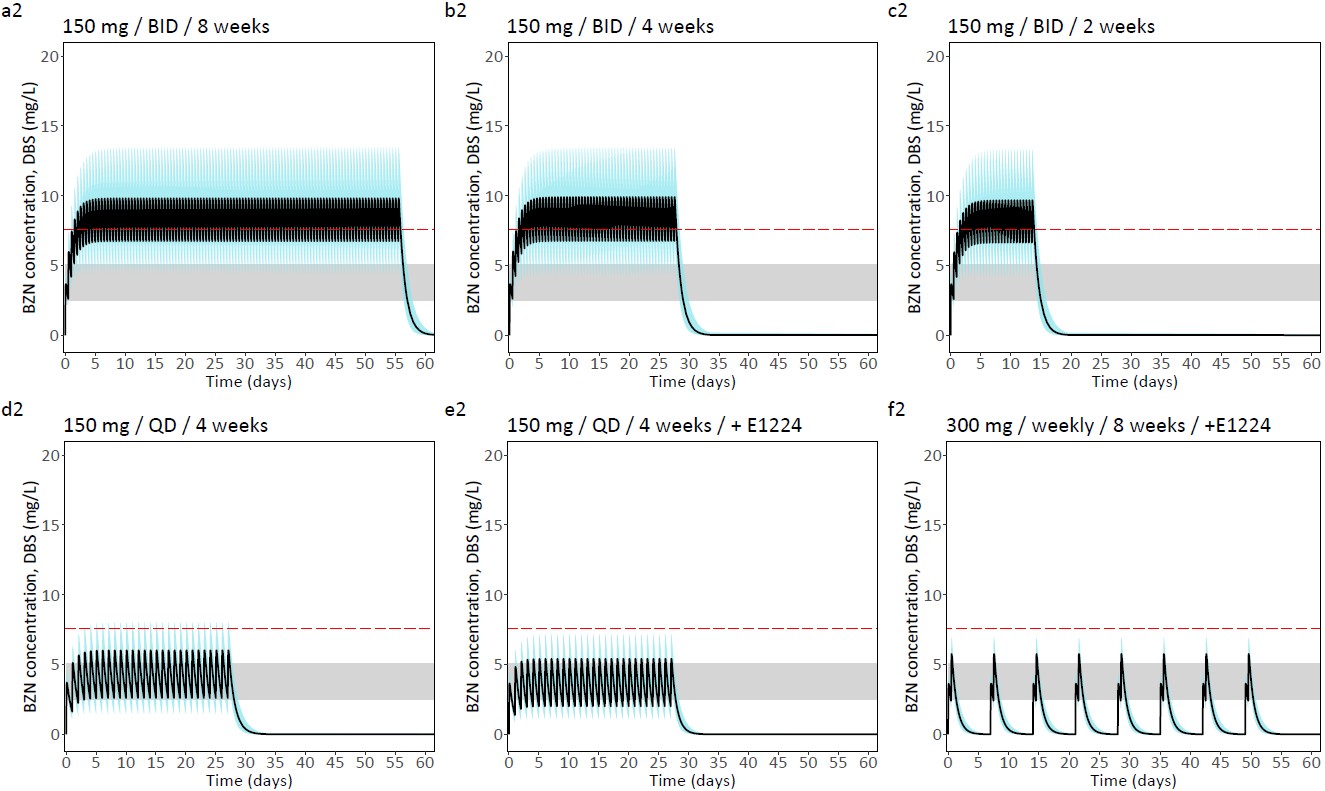 |
| **S3 Fig.** Simulations of pharmacokinetic profiles for benznidazole across the BENDITA treatment arms, shown separately for women (**a1-f1**) and men (**a2-f2**). Simulations were based on the final population PK model, with 1000 patients per treatment arm (body weight: 65 kg). Black solid lines represent the median simulated benznidazole plasma concentrations over time, with the 90% prediction interval shown as shaded area (5th and 95th percentiles). The horizontal red line represents the in vitro IC_90_, corrected for protein binding and scaled to DBS concentrations. The grey shaded area illustrates the historically accepted therapeutic range: 3-6 mg/L in plasma (or 2.5-5 mg/L in DBS). |
