# Supplementary material for: Population pharmacokinetic-pharmacodynamic analysis of benznidazole monotherapy and combination therapy with fosravuconazole in chronic Chagas disease (BENDITA)
Source: PLoS Negl Trop Dis. 2025 Sep 22;19(9):e0013522. doi: 10.1371/journal.pntd.0013522 (PMC12510642; doi:10.1371/journal.pntd.0013522)
Supplement: S4 Fig — (DOCX) [file pntd.0013522.s006.docx]

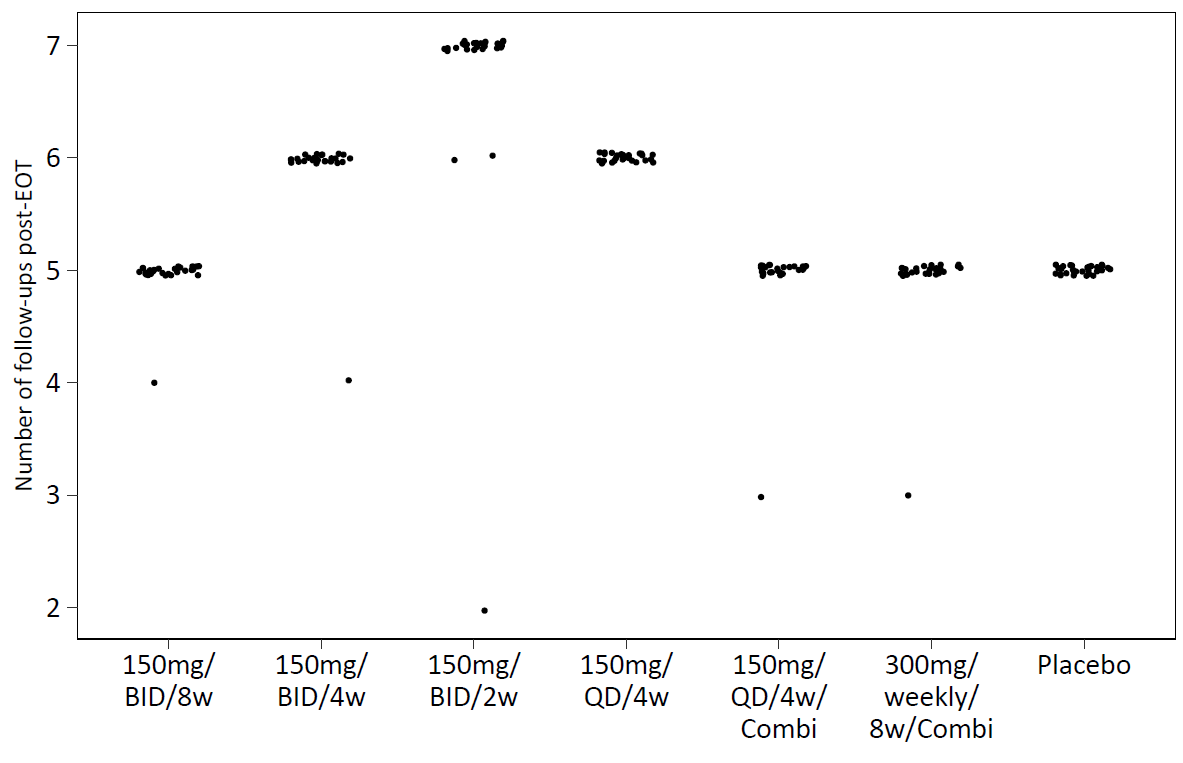


**S4 Fig.** Number of follow-up visits, including end of treatment (EOT) and any follow-up post-EOT, for the different treatment arms in the PK/PD analysis population (n=201). EOT was defined in accordance with the treatment regimen duration, allowing for a two-week grace period after the last active treatment dose.
