# Supplementary material for: Population pharmacokinetic-pharmacodynamic analysis of benznidazole monotherapy and combination therapy with fosravuconazole in chronic Chagas disease (BENDITA)
Source: PLoS Negl Trop Dis. 2025 Sep 22;19(9):e0013522. doi: 10.1371/journal.pntd.0013522 (PMC12510642; doi:10.1371/journal.pntd.0013522)
Supplement: S5 Fig — (DOCX) [file pntd.0013522.s007.docx]

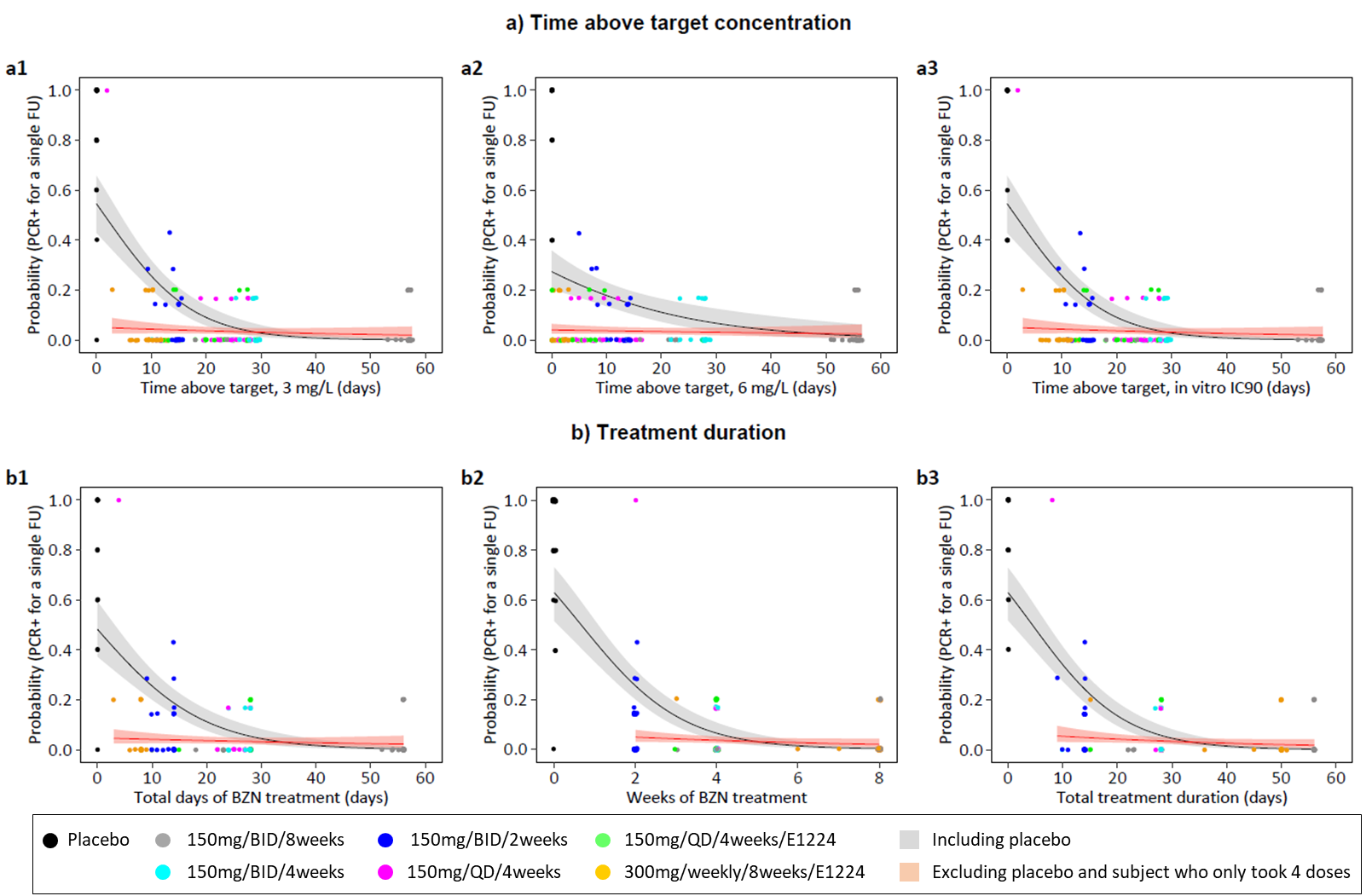


**S5 Fig.** Predicted probabilities of qPCR positivity for a single follow-up visit using different predictors:

**a)** Time above the target concentrations with different thresholds: **a1)** 3 mg/L in plasma, **a2)** 6 mg/L in plasma, and **a3)** scaled in vitro IC_90_;

**b)** Durations of benznidazole treatment using different definitions: **b1)** total days of benznidazole treatment (actual number of days benznidazole was taken), **b2)** weeks of treatment (a week is counted if at least one dose of benznidazole was taken), and **b3)** total treatment duration (regardless of any intermittent interruptions).

The solid grey and red lines indicate the median, and the shaded areas represent the 95% confidence intervals around predicted probabilities. Points represent observed proportions of positive qPCR results after the end of treatment (qPCR positivity) in the placebo arm (black) and active treatment arms.
