# Supplementary material for: Population pharmacokinetic-pharmacodynamic analysis of benznidazole monotherapy and combination therapy with fosravuconazole in chronic Chagas disease (BENDITA)
Source: PLoS Negl Trop Dis. 2025 Sep 22;19(9):e0013522. doi: 10.1371/journal.pntd.0013522 (PMC12510642; doi:10.1371/journal.pntd.0013522)
Supplement: S6 Fig — (DOCX) [file pntd.0013522.s008.docx]

**
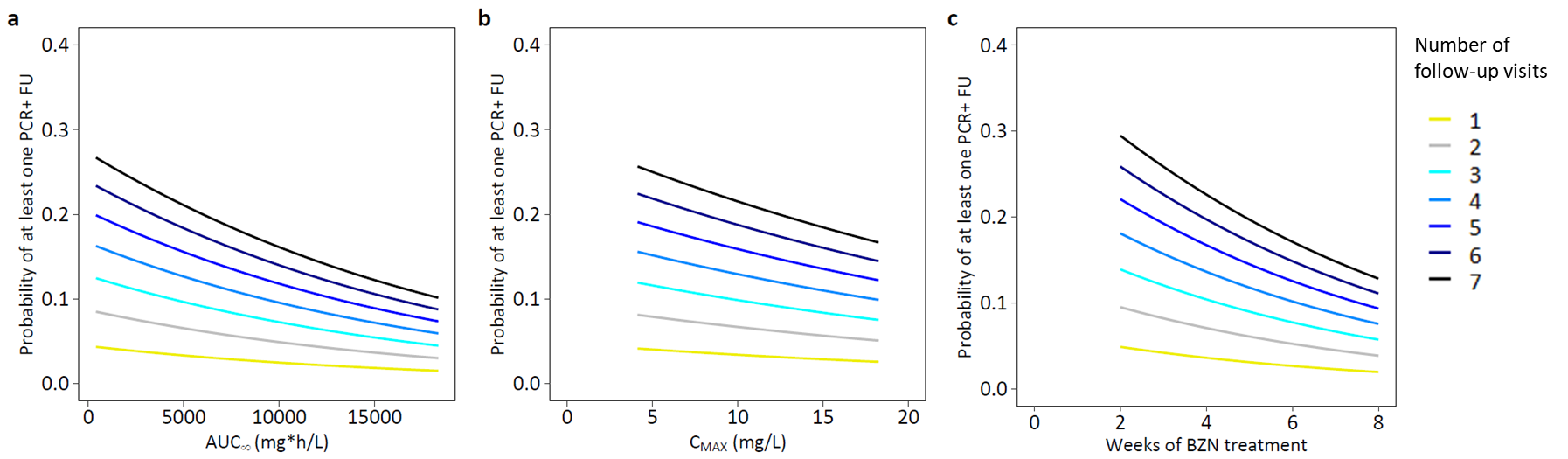
**

**S6 Fig.** Predicted probabilities of at least one follow-up visit being qPCR positive. Probabilities were estimated using binomial regression, based on the modified ITT population (n = 170), excluding placebo and one influential outlier. The lines represent the median predicted probabilities for different numbers of follow-up visits, based on the median Ct value (37.7). 95% confidence intervals are overlapping (not shown).
