# Supplementary material for: Population pharmacokinetic-pharmacodynamic analysis of benznidazole monotherapy and combination therapy with fosravuconazole in chronic Chagas disease (BENDITA)
Source: PLoS Negl Trop Dis. 2025 Sep 22;19(9):e0013522. doi: 10.1371/journal.pntd.0013522 (PMC12510642; doi:10.1371/journal.pntd.0013522)
Supplement: S1 Table — (DOCX) [file pntd.0013522.s009.docx]

**S1 Table.** Secondary pharmacokinetic parameter estimates, based on the final population pharmacokinetic model for benznidazole.

| **Secondary PK parameter** |  | **BZN 300 mg daily for 8 weeks** | **BZN 300 mg daily for 4 weeks** | **BZN 300 mg daily for 2 weeks** | **BZN 150 mg daily for 4 weeks** | **BZN 150 mg daily for 4 weeks + E1224** | **BZN 300 mg once weekly for**  **8 weeks + E1224** |
| --- | --- | --- | --- | --- | --- | --- | --- |
| ***Women*** |  |  |  |  |  |  |  |
| AUC_inf_ (mg×h/L) |  | 12,764  (3,945 - 15,931) | 6,553  (4,963 - 9,491) | 3,207  (1,789 - 4,080) | 2,956  (2,273 - 3,768) | 2,772  (1,433 - 3,946) | 1,506  (978 - 2,139) |
| C_MAX_ (mg/L) |  | 12.7  (10.7 - 14.8) | 12.5  (10.0 - 16.7) | 12.1  (7.1 - 16.3) | 7.2  (5.5 - 8.2) | 6.3  (4.8 - 9.3) | 7.2  (5.4 - 8.9) |
| Time above target (days)^a^ | in vitro IC90 | 48.5  (10.0 - 55.1) | 26.4  (14.5 - 27.8) | 11.3  (0.0 - 13.4) | 0.0  (0.0 - 2.8) | 0.0  (0.0 - 5.8) | 0.0  (0.0 - 1.4) |
|  | 3 mg/L | 57.0  (17.4 - 57.5) | 29.1  (24.9 - 29.7) | 15.0  (11.8 - 15.7) | 25.9  (19.5 - 28.1) | 24.9  (11.8 - 28.1) | 11.0  (6.5 - 14.4) |
|  | 6 mg/L | 55.7  (15.5 - 56.2) | 27.9  (23.0 - 28.6) | 13.8  (7.3 - 14.3) | 10.6  (3.1 - 15.7) | 7.4  (0.0 - 16.0) | 2.5  (0.4 - 5.5) |
| T_MAX_ (hours) |  | 1.77  (1.51 - 4.55) | 1.76  (1.55 - 1.93) | 1.77  (1.50 - 2.05) | 1.99  (1.09 - 2.59) | 2.00  (1.48 - 4.62) | 1.90  (1.43 - 2.76) |
| Terminal t_½_ (hours) |  | 17.8  (14.2 - 19.7) | 17.1  (13.5 - 19.2) | 15.8  (10.5 - 19.7) | 16.1  (13.6 - 19.8) | 14.2  (10.6 - 20.4) | 14.2  (10.7 - 17.6) |
| ***Men*** |  |  |  |  |  |  |  |
| AUC_inf_ (mg×h/L) |  | 10,839  (7,540 - 11,915) | 5,709  (2,498 - 6,462) | 2,593  (1,778 - 3,408) | 2,474  (1,271 - 3,198) | 2,300  (1,784 - 2,796) | 1,304  (960 - 1,435) |
| C_MAX_ (mg/L) |  | 9.9  (7.7 - 10.7) | 10.1  (8.8 - 11.8) | 10.1  (8.6 - 13.1) | 5.9  (4.9 - 7.1) | 5.3  (4.3 - 6.3) | 5.6  (5.0 - 6.6) |
| Time above target (days)^a^ | in vitro IC90 | 34.4  (10.2 - 47.4) | 19.6  (4.3 - 25.8) | 6.1  (2.6 - 12.3) | 0.0  (0.0 - 0.0) | 0.0  (0.0 - 0.0) | 0.0  (0.0 - 0.0) |
|  | 3 mg/L | 56.9  (51.7 - 57.3) | 29.0  (15.2 - 29.6) | 14.8  (9.8 - 15.2) | 22.4  (10.8 - 27.7) | 19.9  (13.6 - 26.6) | 9.1  (6.3 - 10.3) |
|  | 6 mg/L | 55.3  (35.8 - 55.7) | 27.5  (13.4 - 27.9) | 13.2  (8.2 - 13.9) | 4.4  (0.0 - 10.8) | 1.7  (0.0 - 6.8) | 1.2  (0.0 - 1.7) |
| T_MAX_ (hours) |  | 1.80  (1.47 - 2.10) | 1.82  (1.53 - 2.12) | 1.68  (0.49 - 1.96) | 1.82  (1.19 - 2.38) | 1.93  (1.37 - 2.27) | 2.09  (1.76 - 2.43) |
| Terminal t_½_ (hours) |  | 18.6  (13.5 - 19.9) | 17.4  (15.8 - 21.6) | 17.0  (14.5 - 20.0) | 16.8  (14.9 - 20.0) | 14.1  (11.8 - 17.6) | 13.6  (11.7 - 16.5) |
| ***Pooled*** |  |  |  |  |  |  |  |
| AUC_inf_ (mg×h/L) |  | 11,336  (4,153 - 15,561) | 6,275  (2,733 - 8,949) | 3,058  (1,762 - 4,003) | 2,837  (2,213 - 3,746) | 2,601  (1,570 - 3,562) | 1,428  (933 - 2,093) |
| C_MAX_ (mg/L) |  | 11.7  (8.93 - 14.4) | 11.8  (9.01 - 16.1) | 11.9  (7.43 - 15.7) | 6.45  (5.15 - 8.17) | 6.17  (4.51 - 8.69) | 6.92  (5.12 - 8.86) |
| Time above target (days)^a^ | in vitro IC90 | 40.0  (8.92 - 55.0) | 24.6  (6.64 - 27.6) | 10.6  (0.32 - 13.2) | 0.00  (0.00 - 2.47) | 0.00  (0.00 - 2.77) | 0.00  (0.00 - 1.27) |
|  | 3 m/L | 56.9  (20.1 - 57.4) | 29.1  (15.9 - 29.8) | 14.9  (10.7 - 15.7) | 25.4  (19.3 - 28.1) | 22.8  (12.6 - 27.8) | 10.3  (6.2 - 13.9) |
|  | 6 mg/L | 55.4  (17.9 - 56.2) | 27.8  (14.0 - 28.5) | 13.4  (7.7 - 14.3) | 7.7  (0.5 - 15.5) | 5.6  (0.0 - 13.8) | 2.2  (0.1 - 5.2) |
| T_MAX_ (hours) |  | 1.80  (1.47 - 3.72) | 1.77  (1.49 - 2.11) | 1.74  (0.85 - 2.05) | 1.99  (0.96 - 2.45) | 1.97  (1.36 - 4.13) | 1.91  (1.49 - 2.67) |
| Terminal t_½_ (hours) |  | 18.0  (13.8 - 20.0) | 17.1  (13.8 - 20.5) | 16.2  (11.7 - 20.4) | 16.4  (14.0 - 20.5) | 14.2  (11.0 - 19.3) | 13.8  (10.8 - 17.5) |

All values are given as median (5^th^ to 95^th^ percentile). **Abbreviations:** T_MAX_, time after dose to reach the maximum concentration in plasma (C_MAX_); AUC_∞_, cumulative AUC extrapolated to infinity. ^a^Time above target, where the target concentration was either defined as the in vitro IC90, corrected for protein binding to assay medium and plasma protein (see Section S2), or the therapeutically accepted target concentration of 3 to 6 mg/mL benznidazole in plasma. These values were scaled to dry blood spot concentrations using a scaling factor of 0.84, according to Bedor et al (Antimicrob Agents Chemother, 2018. 62(12).
