# Supplementary material for: Population pharmacokinetic-pharmacodynamic analysis of benznidazole monotherapy and combination therapy with fosravuconazole in chronic Chagas disease (BENDITA)
Source: PLoS Negl Trop Dis. 2025 Sep 22;19(9):e0013522. doi: 10.1371/journal.pntd.0013522 (PMC12510642; doi:10.1371/journal.pntd.0013522)
Supplement: S2 Table — (DOCX) [file pntd.0013522.s010.docx]

**S2 Table.** Correlation matrix of plasma variables for benznidazole DBS concentrations in the PK analysis population (n=175).

| **Dosing regimen** | **Cumulative dose (g)** | **AUC_∞_**  **(mgxh/L)** | **C_MAX_**  **(mg/L)** | **T > IC90**  **(days)** | **T > 3 mg/L**  **(days)** | **T > 6 mg/L**  **(days)** | **Days of BZN treatment** | **Weeks of BZN treatment** | **Total duration (days)** |
| --- | --- | --- | --- | --- | --- | --- | --- | --- | --- |
| **Cumulative dose** | 1 |  |  |  |  |  |  |  |  |
| **AUC_∞_** | 0.96 | 1 |  |  |  |  |  |  |  |
| **C_MAX_** | 0.49 | 0.63 | 1 |  |  |  |  |  |  |
| **T > IC90** | 0.92 | 0.96 | 0.71 | 1 |  |  |  |  |  |
| **T > 3 mg/L** | 0.95 | 0.92 | 0.37 | 0.83 | 1 |  |  |  |  |
| **T > 6 mg/L** | 0.97 | 0.96 | 0.62 | 0.95 | 0.92 | 1 |  |  |  |
| **Days of BZN treatment** | 0.91 | 0.86 | 0.25 | 0.75 | 0.97 | 0.84 | 1 |  |  |
| **Weeks BZN treatment** | 0.42 | 0.38 | -0.13 | 0.33 | 0.39 | 0.35 | 0.33 | 1 |  |
| **Total duration** | 0.52 | 0.47 | -0.10 | 0.41 | 0.50 | 0.44 | 0.45 | 0.99 | 1 |

Correlation was assessed using Pearson correlation coefficients (<https://cran.r-project.org/web/packages/corrtable/index.html>), all coefficients were highly significant (p value < 0.001); C_MAX_, maximum plasma concentrations; AUC_∞,_ cumulative area under the concentration-time curve extrapolated to infinity; T>IC_90_, Time above IC_90_ in plasma.
