# Supplementary material for: Population pharmacokinetic-pharmacodynamic analysis of benznidazole monotherapy and combination therapy with fosravuconazole in chronic Chagas disease (BENDITA)
Source: PLoS Negl Trop Dis. 2025 Sep 22;19(9):e0013522. doi: 10.1371/journal.pntd.0013522 (PMC12510642; doi:10.1371/journal.pntd.0013522)
Supplement: S3 Table — (DOCX) [file pntd.0013522.s011.docx]

**S3 Table .** Regression model diagnostics for the correlation between benznidazole exposure and qPCR positivity for subjects meeting the PK/PD reliability criteria (sensitivity analysis).

| **Characteristic** | **Base model** | **AUC_∞_**  **(mgxh/L)** | **Log AUC_∞_**  **(mgxh/L)** | **C_MAX_**  **(mg/L)** | **Time above target (days)^c^** | |  | **Duration of BZN treatment** | | |
| --- | --- | --- | --- | --- | --- | --- | --- | --- | --- | --- |
|  |  |  |  |  | **3 mg/L in plasma** | **6 mg/L in plasma** |  | **Dosing days** | **Weeks ^d^** | **Total duration (days)^e^** |
| **Refined mITT, including placebo and all meeting the PK/PD reliability criteria (n=186)^a^** | | | | | | | | | | |
| Odds ratio, exposure  [95% CI]  p-value |  | 1.000  [0.999, 1.000]  <0.001*** | 0.232  [0.190, 0.285]  <0.001*** | 0.637  [0.575, 0.706]  <0.001*** | 0.889  [0.857, 0.921]  <0.001*** | 0.945  [0.919, 0.972]  <0.001*** |  | 0.908  [0.881, 0.936]  <0.001*** | 0.434  [0.349, 0.540]  <0.001*** | 0.884  [0.857, 0.912]  <0.001*** |
| Odds ratio, Ct value  [95% CI]  p-value | 0.913  [0.806, 1.034]  0.154 | 0.902  [0.797, 1.020]  0.100 | 0.822  [0.724, 0.934]  0.003** | 0.911  [0.810, 1.024]  0.117 | 0.882  [0.780, 0.997]  0.045* | 0.910  [0.802 , 1.032]  0.142 |  | 0.880  [0.777, 0.997]  0.045* | 0.783  [0.683, 0.896]  <0.001*** | 0.781  [0.682, 0.894]  <0.001*** |
| **AIC** | **406.9** | **369.3** | **258.8** | **314.2** | **345.6** | **385.6** |  | **354.0** | **319.3** | **318.2** |
| **Excluding placebo and one subject who took only 4 four BZN doses (n=156)^b^** | | | | | | | | | | |
| Odds ratio, exposure  [95% CI]  p-value | - | 1.0000  [0.9998, 1.0001]  0.528 | 0.546  [0.158, 1.804]  0.328 | 0.963  [0.854, 1.079]  0.526 | 0.991  [0.962, 1.017]  0.521 | 0.994  [0.970, 1.016]  0.622 |  | 0.995  [0.967, 1.020]  0.693 | 0.898  [0.737, 1.070]  0.251 | 0.985  [0.956, 1.011]  0.281 |
| Odds ratio, Ct value  [95% CI]  p-value | 0.918  [0.790, 1.079]  0.282 | 0.9181  [0.7903 1.0785]  0.278 | 0.919  [0.792, 1.079]  0.282 | 0.924  [0.794, 1.087]  0.323 | 0.917  [0.789, 1.077]  0.270 | 0.918  [0.790, 1.079]  0.281 |  | 0.917  [0.790, 1.078]  0.274 | 0.900  [0.769, 1.063]  0.199 | 0.901  [0.770, 1.064]  0.203 |
| **AIC** | **162.9** | **164.5** | **163.9** | **164.5** | **164.4** | **164.6** |  | **164.7** | **163.5** | **163.7** |

**Abbreviations:** mITT, modified intention-to-treat, AIC, Akaice Information Criterion; CI, confidence intervals; Ct, cycle threshold values; *** p < 0.001, ** p < 0.01, * p<0.05; Odds ratios represent the odds ratio of PCR positivity associated with a one-unit increase in the predictor and are presented for various benznidazole exposure metrics and baseline Ct values.

^a^based on beta binomial regression; ^b^based on binomial regression and a dispersion factor of 1; ^c^according to the accepted therapeutic range: 3-6 mg/L in plasma (2.5-5 mg/L in DBS); ^d^ a week is counted if at least one benznidazole dose per week was taken
